# Secretion of the cytoplasmic and high molecular weight *β*-galactosidase of *Paenibacillus wynnii* with *Bacillus subtilis*

**DOI:** 10.1186/s12934-024-02445-7

**Published:** 2024-06-12

**Authors:** Jana Senger, Ines Seitl, Eva Pross, Lutz Fischer

**Affiliations:** Institute of Food Science and Biotechnology, Department of Biotechnology and Enzyme Science, University of Hohenheim, Garbenstr. 25, 70599 Stuttgart, Germany

**Keywords:** *Bacillus subtilis*, Protein secretion, Recombinant enzyme production, *β*-galactosidase, Bioreactor

## Abstract

**Background:**

The gram-positive bacterium *Bacillus subtilis* is widely used for industrial enzyme production. Its ability to secrete a wide range of enzymes into the extracellular medium especially facilitates downstream processing since cell disruption is avoided. Although various heterologous enzymes have been successfully secreted with *B. subtilis*, the secretion of cytoplasmic enzymes with high molecular weight is challenging. Only a few studies report on the secretion of cytoplasmic enzymes with a molecular weight > 100 kDa.

**Results:**

In this study, the cytoplasmic and 120 kDa *β*-galactosidase of *Paenibacillus wynnii* (*β*-gal-Pw) was expressed and secreted with *B. subtilis* SCK6. Different strategies were focused on to identify the best secretion conditions. Tailormade codon-optimization of the *β*-gal-Pw gene led to an increase in extracellular *β*-gal-Pw production. Consequently, the optimized gene was used to test four signal peptides and two promoters in different combinations. Differences in extracellular *β*-gal-Pw activity between the recombinant *B. subtilis* strains were observed with the successful secretion being highly dependent on the specific combination of promoter and signal peptide used. Interestingly, signal peptides of both the general secretory- and the twin-arginine translocation pathway mediated secretion. The highest extracellular activity of 55.2 ± 6 µkat/L_culture_ was reached when secretion was mediated by the PhoD signal peptide and expression was controlled by the P_AprE_ promoter. Production of extracellular *β*-gal-Pw was further enhanced 1.4-fold in a bioreactor cultivation to 77.5 ± 10 µkat/L_culture_ with secretion efficiencies of more than 80%.

**Conclusion:**

For the first time, the *β*-gal-Pw was efficiently secreted with *B. subtilis* SCK6, demonstrating the potential of this strain for secretory production of cytoplasmic, high molecular weight enzymes.

**Supplementary Information:**

The online version contains supplementary material available at 10.1186/s12934-024-02445-7.

## Background

*β*-galactosidases (EC 3.2.1.23) catalyze the hydrolysis of lactose into D-galactose and D-glucose and are widely applied in the dairy industry for the production of lactose-free dairy products [1, 2]. In addition, *β*-galactosidases are used for the transglycosylation of lactose for the synthesis of galactooligosaccharides, which serve as prebiotics [3]. The *β*-galactosidase of *Paenibacillus wynnii* (*β*-gal-Pw) is a 120 kDa enzyme which has favorable kinetic properties for the production of lactose-free and galactooligosaccharide-enriched dairy products. In comparison to other industrial-relevant *β*-galactosidases, *β*-gal-Pw has a high substrate affinity for lactose (*K*_M,__Lactose_ = 0.63 ± 0.045 mM in milk), is not inhibited by the D-galactose product formed and completely hydrolyzes lactose in 72 h at 8 °C. In addition, the *β*-gal-Pw showed a long half-life of 77 days at 8 °C [4, 5].

So far, *β*-gal-Pw has only been produced intracellularly using *Escherichia coli* BL21 [4, 5]. Intracellular enzyme production leads to high expression levels but often requires a time-consuming and costly downstream processing, such as cell disruption and lysate purification. By contrast, secretory enzyme production circumvents these additional steps since the enzyme can be directly purified from the supernatant [6, 7]. Most industrial enzymes are produced by secretion, such as proteases, cellulases or amylases [6]. Nevertheless, the secretion of heterologous enzymes is a complex mechanism including many crucial steps, such as expression, folding, secretion and extracellular processing [8, 9]. Therefore, the choice of an appropriate production host is decisive. Various factors, such as production speed, protein quality, functionality and yield, affect the choice of the production host [7]. Depending on the target protein, different eukaryotic hosts, for example, molds and yeasts, as well as bacteria are often used for secretory production. The gram-positive bacterium *Bacillus subtilis* is a prokaryotic host widely used for secretory enzyme production. Its qualified presumption of safety status granted by the European Food Safety Authority makes this microbe an especially attractive host for the production of food and feed enzymes [10]. In addition, *B. subtilis* is genetically well investigated and secretes enzymes at up to g/L scale [11]. Two routes are mainly used for secretion: the translocation of unfolded protein is facilitated by the general secretory (Sec) pathway, whereas the secretion of folded proteins takes place via the twin-arginine translocation (TAT) pathway. Both pathways require a signal peptide fused to the N-terminus of the protein, which is cleaved during the translocation across the cell membrane by signal peptidases [12–14]. Predominantly homologous enzymes are industrially produced with *Bacillus* species [7]. However, many heterologous enzymes were secreted by fusing a *Bacillus* signal peptide to the enzyme of interest, for example, *β*-galactosidases, cutinases or phytases [12, 15–17]. A lot of effort has been spent to improve the secretory production yield, including the screening and modification of expression elements [18, 19], signal peptides [16, 20] and cultivation strategies [21]. Furthermore, the production was increased when extracellular proteases were deleted or chaperones were overexpressed [22, 23]. However, the secretion of heterologous enzymes is often limited due to several bottlenecks during translocation, post-translational folding or extracellular proteolysis [24–26]. The secretion of natively cytoplasmic enzymes with a high molecular weight seems to be especially challenging. Only three studies to date have reported the secretion of a cytoplasmic enzyme with a molecular weight higher than 100 kDa with *B. subtilis*, among them the *β*-galactosidases of *Bacillus megaterium* and *Escherichia coli* with molecular weights of 119 kDa and 116 kDa [27–29], respectively. Both studies fused a *Bacillus* signal peptide, either WapA or AmyE, to the gene of interest and reached extracellular activities of 17.55 U/mL (= 292.5 µkat/L) and 310 U/mL (= 5167 µkat/L) for the *β*-galactosidases of *B. megaterium* and *E. coli*, respectively [27, 29].

In this study, the secretion of the high molecular weight *β*-gal-Pw was investigated in *B. subtilis* SCK6. This strain is often used for secretory enzyme production [30–33] because it is deficient in two major extracellular proteases, AprE and NprE, which reduce the extracellular protease activity significantly [34]. In addition, a second gene copy of the competence transcription factor *comK* was integrated into the genome of *B. subtilis* SCK6 under the control of a xylose-inducible promoter, which improves the transformation efficiency [35]. Two promoters in combination with four signal peptides were tested for secretory *β*-gal-Pw production. Therefore, the widely used promoters P_43_ and P_AprE_, which facilitate efficient expression in several studies [e.g. 20, 36–38], were compared. Furthermore, different signal peptides directing secretion via the Sec or TAT pathway were fused to the N-terminus of the *β*-gal-Pw protein. The screening of promoter signal peptide combinations and further scale-up of cultivation should evaluate the potential of *B. subtilis* for the secretory production of the cytoplasmic and high molecular weight enzyme *β*-gal-Pw and provide a basis for further optimizations.

## Methods

### Chemicals and enzymes

Chemicals in this study were purchased from Carl Roth GmbH (Karlsruhe, Germany), Sigma Aldrich (St. Louis, USA), Biosolve (Valkenswaard, Netherlands) and Fisher Scientific (Hampton, USA). Oligos were ordered from Biomers (Ulm, Germany). Enzymes for cloning, T4 Polynucleotide Kinase and Q5® High-Fidelity DNA Polymerase for polymerase chain reaction were obtained from New England Biolabs GmbH (NEB; Germany). T4 DNA ligase was purchased from Thermo Fisher Scientific (Hampton, USA).

### Strains and media

*E. coli* XL1 was grown in Luria-Bertani (LB) medium at 37 °C containing 100 µg/mL ampicillin. *B. subtilis* SCK6 [35] strains were cultivated in LB medium at 37 °C or S1 medium for recombinant *β*-gal-Pw production. S1 medium consisted of part 1 (10 g/L soytone) and part 2 (75 g/L glucose, 3.6 g/L Urea, 3 mL/L 1 M K_2_HPO_4_, 100 mL 10 x MOPS minerals). The pH of both parts was adjusted to 7.3 and part 1 was sterilized by autoclaving and part 2 by filtration (Ø 0.2 μm). The 10 x MOPS minerals were comprised of 83.7 g/L MOPS, 7.2 g/L Tricine, 12 g/L KOH, 29.3 g/L NaCl, 450 µL/L 0.5 M K_2_SO_4_, 10 mL/L 0.5 M MgCl_2_, 100 mL/L 100 × Micronutritions (1.5 g/L Na_3_Citrate × 2 H_2_O, 1.5 g/L CaCl_2_ × 2 H_2_O, 0.4 g/L FeSO_4_ × 7 H_2_O, 0.1 g/L MnSO_4_ × H_2_O, 0.16 g/L ZnSO_4_ × 7 H_2_O, 0.056 g/L CuCl_2_ × 2 H_2_O, 0.1 g/L CoCl_2_ × 6 H_2_O, 0.1 g/L Na_2_MoO_4_ × 2 H_2_O). An amount of 7.5 µg/mL neomycin was added for selection.

### Construction of a modular expression cassette

**Fig. 1:**
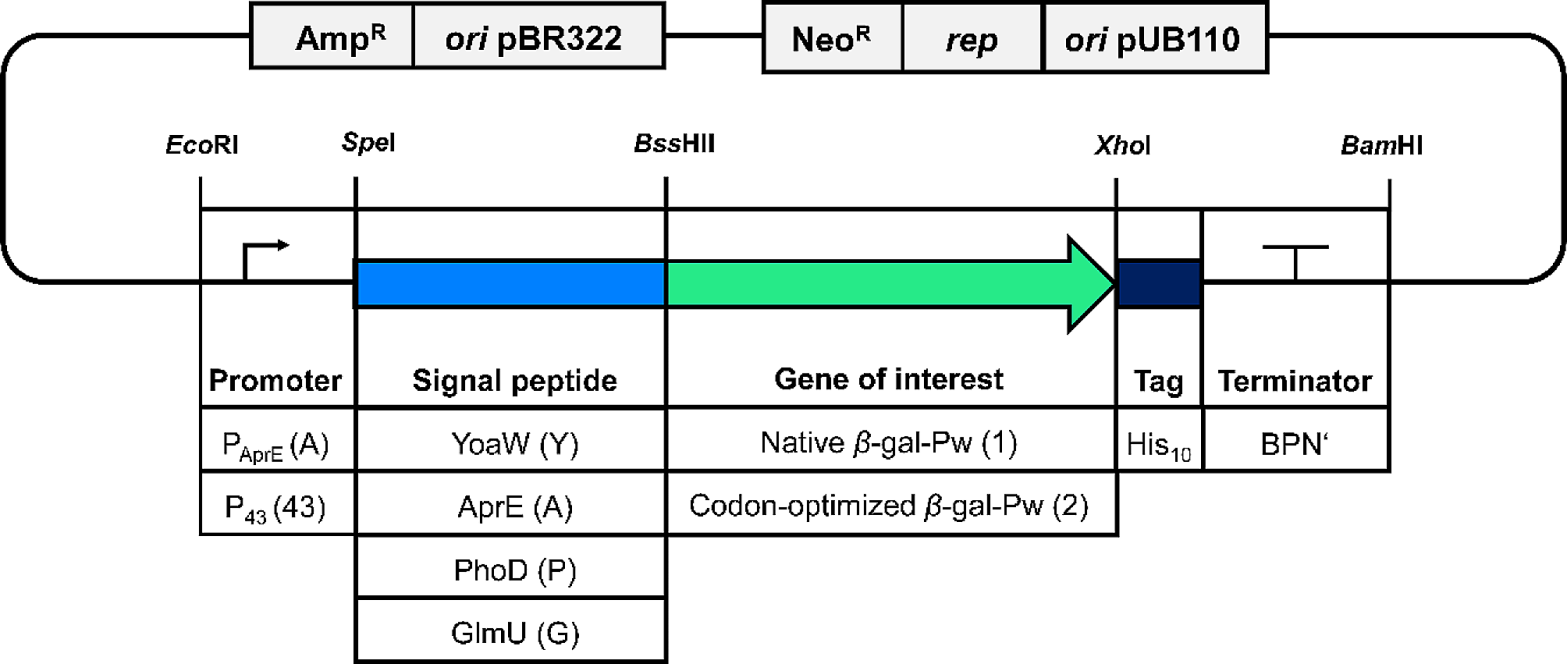
Schematic overview of generated plasmids with a modular expression cassette. Plasmids were generated to test different combinations of expression and secretion elements. Symbols in brackets refer to the abbreviation used for recombinant strain designation

Primers, signal peptides, plasmids and strains used and generated in this study are listed in Table S1, S2, S3 and S4, respectively (Additional File 1). All plasmids generated were shuttle plasmids based on the pLF vector backbone provided on plasmid BNspeBif3. It possesses the ColE1 *ori* for replication in *E. coli* and pUB110 *ori* with the *rep* gene for rolling circle replication in *B. subtilis*. Ampicillin- and neomycin-resistance genes were present for selection in *E. coli* and *B. subtilis*, respectively. A modular expression cassette was designed and synthesized (Invitrogen, Thermo Fisher Scientific). It was composed of the AprE signal peptide, cloning sites for the gene of interest, a C-terminal His_10_-tag and the terminator region of subtilisin (BPN’) of *Bacillus amyloliquefaciens.* The restriction sites in the expression cassette enabled the exchange of the promoter, signal peptide, gene of interest and terminator by conventional cloning via restriction and ligation (Fig. 1). The cassette was integrated into the pLF vector by *Bam*HI and *Eco*RI. Exchange of the promoter and signal peptide was possible by *Eco*RI/*Spe*I and *Spe*I/*Bss*HII digestion, respectively. Integration of the gene of interest was done via *Bss*HII/*Xho*I or *Bsm*BI/*Xho*I digestion. *Bsm*BI was used due to a *Bss*HII restriction site in the native *β*-gal-Pw sequence. Insert and vector were digested and Shrimp Alkaline Phosphatase (rSAP; NEB, Germany) was added for the vector sample. The samples digested were purified from 1% (w/v) agarose gels after agarose gel electrophoresis (AGE) using the GeneJET Gel-Extraction-Kit (Thermo Fisher Scientific, Hampton, USA). Vector and insert were ligated at a molar ratio of 1:4 with T4 DNA ligase for 1 h at room temperature. The sample was used for the transformation of *E. coli* XL1. The plasmid was isolated from single colonies of *E. coli* XL1 using the GeneJET Plasmid-Miniprep-Kit (Thermo Fisher Scientific, Hampton, USA). Correct cloning was verified by digestion and sequencing (Eurofins Genomics, Ebersberg, Germany). Genomic DNA of *B. subtilis* 168 was isolated using a GeneJET genomic DNA Purification Kit (Thermo Fisher Scientific, Hampton, USA) and subsequently used as a template for the amplification of the P_43_ promoter region with primers P3 and P4 (Additional File 1: Table S1). The amplified fragment was purified using the DNA clean & concentrator Kit (Zymo Research, Orange, CA) and integrated into the pLF vector via *Eco*RI/*Spe*I.

The YoaW signal peptide was designed as oligonucleotides O1 and O2 with *Spe*I and *Bss*HII 5’ overhangs. Firstly, the oligonucleotides were phosphorylated separately using T4 Polynucleotide Kinase (T4 PNK) for 1 h at 37 °C. After heat inactivation of T4 PNK for 20 min at 65 °C, the oligonucleotides were annealed using the following conditions: (i) 120 s at 95 °C; (ii) 70 cycles for 45 s at 94 − 25 °C with a 1 °C decrease in each cycle step and, (iii) cooling at 4 °C. Due to longer sequences, the GlmU and PhoD signal peptides were designed with *Spe*I 5’ and *Bss*HII 3’ overhang and synthesized by Invitrogen.

The native *β*-gal-Pw gene was amplified from a plasmid using the Q5 DNA polymerase and primers P1 and P2 (Additional File 1: Table S1), and the codon-optimized sequence was synthesized by Invitrogen. Primers and sequence were designed for integration into the pLF_P_AprE_ vector via *Bsm*BI/*Xho*I or *Bss*HII/*Xho*I digestion. Therefore, a *Xho*I restriction site was integrated at the 3’ end *β*-gal-Pw gene. Regarding the amplified, native *β*-gal-Pw sequence, *Bsm*BI was inserted at the 5’ end of the gene due to a *Bss*HII site in the gene sequence. The codon-optimized *β*-gal-Pw sequence was synthesized with a *Bss*HII site at the 5’ end of the sequence. Due to a *Bss*HII site in the P_43_ promoter, integration into the pLF_P_43_ vectors was done based on pLF_P_AprE_ constructs by S*pe*I/*Xho*I digestion. In that way, the signal peptide-gene fragment was exchanged.

### Transformation of *B. subtilis* SCK6

The transformation of *B. subtilis* SCK6 was done as described previously [35], with some modifications. An amount of 3 mL LB medium was inoculated with a single colony of *B. subtilis* SCK6 and cultivated overnight at 37 °C and 180 rpm. The culture was diluted to an OD_600_ of 0.8 using LB medium with 1% D-xylose (w/v) and further incubated for 2 h at 37 °C and 180 rpm to induce *comK* expression. Plasmid DNA was amplified using the Cytiva IllustraTM TempliphiTM kit (Fisher Scientific, Hampton, USA) and 1 µg of DNA were added to 200 µL of competent *B. subtilis* SCK6 cells. After incubation for 1 h at 37 °C and 200 rpm, cells were plated on LB agar plates with 7.5 µg/mL neomycin and 80 µg/mL X-Gal and incubated overnight at 37 °C or for two days at 30 °C.

### Shake flask cultivation of recombinant *B. subtilis* strains

Recombinant *B. subtilis* strains were screened by shake flask cultivation. Therefore, freshly transformed *B. subtilis* cells were used for the inoculation of 10 mL S1 medium with 7.5 µg/mL neomycin and incubated for 14–18 h at 37 °C and 180 rpm. The pre-cultures were used for the inoculation of the main cultures with a starting OD_600_ of 0.05. Cultivation was done in triplicate in a 1 L shake flask with 200 mL S1 medium and 7.5 µg/mL neomycin at 30 °C and 110 rpm for 73 h. Sampling was performed periodically for the determination of OD_600_, pH, protein content and extracellular *β*-galactosidase activity.

### Bioreactor cultivation of *Bs*AP2

Batch cultivation was done in duplicates in 1 L Multifors bioreactor systems (Infors, HT) with a working volume of 800 mL. Temperature and aeration rate were kept at 30 °C and 1.5 vvm, respectively. The pH was not regulated and pO_2_ was held above 30% by increasing the stirrer speed stepwise. 405-DPAS-SC-K8S pH and InPro 6900 sensors of Mettler Toledo were used for the detection of pH and pO_2_, respectively. S1 medium with 7.5 ng/µL neomycin was used for fermentation. If required, Antifoam 204 (Sigma Aldrich, USA) was added as antifoaming reagent.

S1 medium with 7.5 ng/µL neomycin was used for preparation of pre-cultures. An amount of 10 mL in 100 mL shaking flasks was inoculated with freshly transformed *B. subtilis*. After incubation at 37 °C at 180 rpm for 13 h, it was used for the inoculation of 100 mL medium in 1 L flasks and incubated for 9.5 h at 37 °C and 110 rpm. An amount of 80 mL of pre-culture was used to inoculate 720 mL of medium in the bioreactor. Samples were taken periodically and OD_600_, bio dry mass and intra- and extracellular *β*-galactosidase activity were determined. All measurements were done in triplicate.

### Sample preparation and determination of protein concentration

Regarding the determination of enzyme activity, the cells were removed from the culture broth by centrifugation at 8000 × g and 4 °C for 10 min. An amount of 1 mL of the supernatant was loaded onto a PD midiTrap G-25 column (Cytiva, MA, USA) and eluted with 1.5 mL activity buffer (100 mM potassium phosphate, pH 6.75 with 5 mM MgCl_2_). Cell pellets were washed with 0.9% (w/v) NaCl and a 30% cell suspension was prepared using activity buffer with 2 mg/mL lysozyme and 1 mM phenylmethylsulfonyl fluoride. After incubation at room temperature for 1 h, the cells were disrupted mixing 1.5 g of 0.1–0.2 mm glass beads (Willy A. Bachofen GmbH, Switzerland) and 1.5 mL cell suspension in the TissueLyser II (Qiagen, Hilden, Germany) for 30 min and 30 Hz. Cell debris was removed by centrifugation at 13,000 rpm for 10 min at 4 °C. Supernatant and cell-free extract were used for the determination of *β*-galactosidase activity and protein concentration according to Bradford [39], using bovine serum albumin as a standard.

### Concentration and purification of *β*-gal-Pw from culture supernatant

The supernatant of the reactor cultivations was centrifuged again at 13,000 rpm for 20 min at 4 °C to remove any remaining cells. An amount of 1 mM phenylmethylsulfonyl fluoride was added to prevent protease degradation. The supernatant was concentrated using VivaFlow® 50 (Sartorius, Göttingen). The cassettes were washed with ddH_2_O and equilibrated with buffer (100 mM potassium phosphate buffer, pH 6.75 with 5 mM MgCl_2_). Concentration was done at 4 °C until a 10-fold concentration was reached. Afterwards, 20 mM imidazole and NaCl were added to a final concentration of 300 mM. The supernatant was centrifuged for 30 min at 13,000 rpm at 4 °C. The *β*-gal-Pw in the concentrated supernatant was purified by immobilized metal affinity chromatography (IMAC) using the Äktapurifier system UPC100 (GE Healthcare, Illinois, USA) and 1 mL HisTrap HP His tag protein purification columns (Cytiva, MA, USA). Column equilibration was done with binding buffer (20 mM potassium phosphate buffer, 5 mM MgCl_2_, 300 mM NaCl, 20 mM imidazole, pH 7.0). After a wash step for 5 CV using binding buffer, elution was done using a linear gradient of 20 CV to a final concentration of 100% gradient with elution buffer (20 mM potassium phosphate buffer, 5 mM MgCl_2_, 300 mM NaCl, 500 mM imidazole, pH 7.0). An amount of 370 mg of protein was loaded into a volume of 49.5 mL. The fractions collected were loaded onto PD midiTrap G-25 columns (Cytiva, MA, USA) and eluted using activity buffer (100 mM potassium phosphate buffer, 5 mM MgCl_2_, pH 6.75). Samples were analyzed for protein content and *β*-galactosidase activity.

### Size exclusion chromatography

Size exclusion chromatography was done using the Superdex 200 10/300 GL column (Cytiva, Fisher Scientific, Hampton, USA) and the Äktapurifier system UPC100 (GE Healthcare, Illinois, USA). The IMAC-purified sample was concentrated using Vivaspin 6 Centrifugal Concentrators (30 kDa cutoff; Sartorius, Göttingen, Germany), filtered (Ø 0.2 μm) and 500 µL was loaded onto the column. Purification was done with an isocratic run using SEC buffer (50 mM Potassium phosphate buffer, 5 mM MgCl_2_, 300 mM NaCl, pH 6.75) and a flow rate of 0.5 mL/min. Molecular weight was calculated using high molecular weight standards of the GE Healthcare Gel filtration calibration kit (Illinois, USA).

### Determination of *β*-galactosidase activity

The *β*-galactosidase activity was determined as described previously [40], using *o*NPG (*o*-nitrophenyl-*β*-D-galactopyranoside) as substrate. An amount of 20 µL of buffer-exchanged supernatant or cell-free extract was added to a mixture of 80 µL buffer (100 mM potassium phosphate buffer, pH 6.75 with 5 mM MgCl_2_) and 100 µL 50 mM *o*NPG dissolved in buffer. The mixture and enzyme solution were pre-incubated separately for 5 min at 37 °C and 900 rpm. The reaction was started by adding the enzyme solution and stopped when the sample turned yellow by the addition of 1 M Na_2_CO_3_. One katal was defined as the amount of enzyme that catalyzes the release of 1 mol *o*-nitrophenol from *o*NPG per second. The enzyme activity of each sample was determined in triplicate.

### SDS-PAGE analysis of *B. subtilis* secretome

The secretome and intracellular proteome of *B. subtilis* strains were visualized by SDS PAGE using a 8% separating gel [41]. An amount of 8 µg of protein was loaded onto the gel either directly from the buffer-exchanged supernatant or after trichloroacetic acid precipitation. Regarding trichloroacetic acid precipitation, a sample volume corresponding to 8 µg of protein was mixed with 15% (w/v) trichloroacetic acid. The sample was incubated at -20 °C for 20 min or for 1 h on ice and centrifuged at 14,000 rpm and 4 °C for 10 min. After washing the pellet with 100% ice-cold acetone and repetition of the centrifugation step, the pellet was dried at 95 °C and solved in 10 µL of 1 × SDS sample buffer (0.02% (w/v) Tris-HCl, 6% (w/v) glycerol, 0.1% (w/v) bromophenol blue, 4% (w/v) SDS and 2% (w/v) *β*-mercaptoethanol). An amount of 5 µL protein molecular weight marker Color Precision Plus Protein™ Unstained protein standard (1,610,363, Bio-Rad, CA, USA) was used as reference. Proteins were visualized with Coomassie Brilliant Blue G-250 staining [42].

### In-gel digest

Proteins were digested in-gel using trypsin (Roche, Germany), according to Shevchenko et al. [43], with some exceptions. After the dehydration of the gel piece with acetonitrile and the reduction of proteins with 10 mM dithiothreitol in 10 mM NH_4_HCO_3_ for 30 min at 56 °C, the dithiothreitol solution was replaced with 55 mM Chloracetamid (Sigma Aldrich, USA). After 20 min incubation in the dark, the gel piece was washed with 200 µL of 100 mM NH_4_HCO_3_, dehydrated with 150 µL of acetonitrile and swollen in 40 mM NH_4_HCO_3_ with 10 ng/µL trypsin on ice for 30 min. Incubation was done overnight at 37 °C and the reaction was stopped by addition of 2 µL of 10% trifluoroacetic acid per 25 µL supernatant. The supernatant was removed and 100 µL 66% acetonitrile with 1.7% acetic acid were added to the gel pieces for elution. After incubation for 15 min at 37 °C, the supernatant was dried in the vacuum centrifuge and the dried samples were resuspended in 20 µL of 0.1% trifluoroacetic acid.

### NanoLC-MS/MS analysis

Nano-LC-ESI-MS/MS experiments were performed on either (I) an Ultimate 3000 RSLCnano system (Dionex) coupled to an Orbitrap Exploris 480 mass spectrometer using an EASY-Nano Flex source or (II) an EASY-nLC 1200 system coupled to a Q Exactive HF mass spectrometer using an NanosprayFlex source. All systems, mass spectrometers and sources were from Thermo Fisher Scientific (Germany). Experiments were done in the MS Core Facility module (University of Hohenheim, Stuttgart, Germany). Tryptic peptides of system (I) were directly injected into a precolumn (µ-precolumn C18 PepMap100, 300 μm, 100 Å, 5 μm x 5 mm, Thermo Fisher Scientific) and an analytical column (NanoEase M/Z HSS C18 T3, 1.8 μm 100 Å 75 μm x 250 mm column, Waters GmbH, Germany) operated at a constant temperature of 35 °C. Tryptic peptides of system (II) were injected directly into the analytical column. Gradient elution was performed at a flow rate of 300 (I) or 250 nL/min (II) using a 30 min gradient with the following profile: 2–5% solvent B in 30 min, 55–95% solvent B in 10 min, 5 min isocratic at 95% solvent B, then re-equilibration for 10 min from 95 to 2% B and isocratic flow with 2% B for 5 min. Solvents used were 0.1% formic acid (solvent A) and 0.1% formic acid in 80% acetonitril (solvent B). The Orbitrap Exploris 480 was operated under the control of XCalibur software (version 4.4.) (Thermo Fisher Scientific Inc., USA), whereas the Q Exactive HF was operated under the control of XCalibur software (version 4.0.) (Thermo Fisher Scientific Inc., USA). Internal calibration was performed using lock-mass ions from ambient air, as described in Olsen et al. Survey spectra (m/z = 200–2000) were detected in the Orbitrap at a resolution of 60,000 at m/z = 200. Data-dependent MS/MS mass spectra were generated for the 30 most abundant peptide precursors in the Orbitrap using high energy collision dissociation fragmentation at a resolution of 15,000 with normalized collision energy of 30 (I) or 27 (II).

### MS data analysis

Mascot 2.6 (Matrix Science, UK) was used as a search engine for protein identification. Spectra were searched against the *B. subtilis* database from Uniprot (https://www.uniprot.org/, February 2022) with custom-specific protein sequence downloaded as FASTA-formatted sequences. Search parameters specified enzyme none, allowing no missed cleavages, a 5 ppm mass tolerance for peptide precursors and 0.02 Da tolerance for fragment ions. Methionine oxidation was allowed as a variable modification and the carbamidomethylation of cysteine residues was set as a fixed modification. The Mascot results were transferred to Scaffold™ Software 4.10.0 (Proteome Software, USA).

### *In silico* and statistical analyses

The translation initiation rate was calculated using the RBS Calculator v2.1 [44]. Excel was used for calculations and standard deviation was used for data evaluation. Significance analyses were performed using SPSS. All experiments were performed at least in biological duplicates, with three independent measurements.

## Results

### Cloning of expression plasmids for recombinant *B. subtilis* strains

A modular cassette was designed and integrated into the pLF vector. The latter is a shuttle vector for the replication and selection in *E. coli* via the pBR322 *ori* and ampicillin resistance, and in *B. subtilis* via the pUB110 *ori* and neomycin resistance. The cassette possessed a multiple cloning site, which enabled the simple exchange of the promoter, signal peptide, gene of interest and terminator (Fig. 1). In this way, different combinations for expression and secretion were tested by exchange of the modules.

Two well-described promoters P_AprE_ and P_43_ [45, 46], were integrated into the cassette via *Eco*RI/*Spe*I. Together with the BPN’ terminator of *B. amyloliquefaciens*, they enabled the transcription of the gene of interest. The promoters were tested in combination with four different signal peptides. The YoaW (Y) and AprE (A) signal peptides originate from *B. subtilis* and direct secretion via the Sec pathway. YoaW is from an unknown protein whereas AprE is the signal peptide of the major extracellular serine protease AprE (subtilisin) and has often been used for the secretion of heterologous enzymes in *Bacillus* [20, 47, 48]. However, most studies showed the secretion of *β*-galactosidases by using TAT signal peptides [15, 29, 49]. Therefore, the PhoD (P) signal peptide of *B. subtilis* phosphodiesterase and the GlmU (G) signal peptide of *Bacillus licheniformis*, which are described to mediate secretion via the TAT pathway [50, 51], were tested. The signal peptides were integrated into the cassette by *Spe*I/*Bss*HII. Either the native (1) or codon-optimized *β*-gal-Pw gene sequence (2) was used for expression and integrated in-frame with the N-terminal signal peptide and C-terminal His_10_-tag. The expression plasmids constructed (Additional File 1: Table S3) were used for the generation of recombinant *B. subtilis* strains. The recombinant *B. subtilis* SCK6 strains (*Bs*) were named after the respective combination of the promoter (A or 43), signal peptide (Y, A, P or G) and gene sequence (1 or 2) (Additional File 1: Table S4).

### Codon optimization of *β*-gal-Pw gene

*B. subtilis* does not have a strong codon bias [52]. Nevertheless, depending on the biological function, genes in *B. subtilis* can be clustered into three well separated classes, which show differences in codon preference [53]. Class I genes are maintained at a constitutively low level and have a weak codon bias. By contrast, class II genes, which are expressed at high levels in the exponential growth phase, show a strong codon bias [53]. The P_43_ and P_AprE_ promoters natively control expression of the cytidine deaminase (*cdd*) and the subtilisin (*aprE*) genes, that are mainly expressed at the transition and early stationary phase [45, 46]. The codon preferences of these two genes were analyzed. The *aprE* codon bias especially fits to the class II genes described by Moszer et al. [53]. Based on this class II codon bias, the *β*-gal-Pw gene sequence was codon-optimized by exchanging 61 of the rare codons (Additional File 1: Figure S1).

**Fig. 2:**
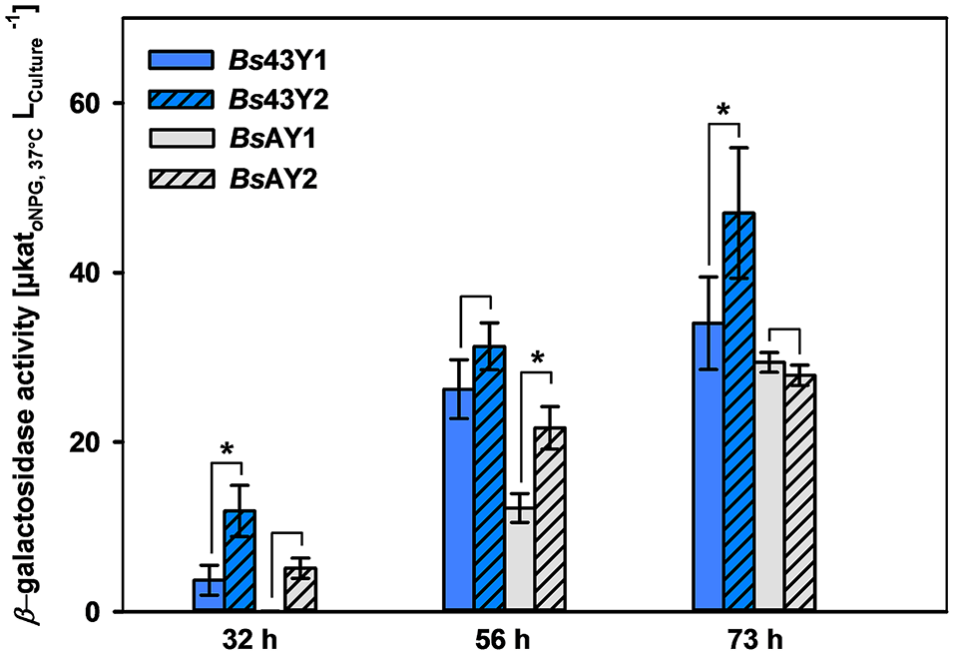
Comparison of native and codon-optimized *β*-gal-Pw gene sequence for extracellular production. Expression of the native (*Bs*43Y1 and *Bs*AY1) or codon-optimized (*Bs*43Y2 and *Bs*AY2) gene was under control of the P_43_ (blue) and P_AprE_ (grey) promoter. Shake flask cultivation was done for 73 h with sampling after 32, 56 and 73 h of cultivation. The star (*) indicates significance (*p* < 0.05)

After synthesis, the sequence was integrated into the modular cassette with the YoaW signal peptide under control of the two promoters yielding *Bs*43Y2 and *Bs*AY2. The impact of the native and optimized sequence was compared in shake flask cultivations. *Bs*43Y2 produced an enhanced extracellular *β*-galactosidase activity due to the codon optimization from 34.0 ± 6 to 47.0 ± 8 µkat/L after 73 h compared to the native *β*-gal-Pw gene in *Bs*43Y1 (Fig. 2). However, this was not the same for the P_AprE_ promoter, where no differences between native (*Bs*AY1) and optimized sequence (*Bs*AY2) were observed after 73 h. However, the tailormade codon optimization of the *β*-gal-Pw gene seemed to improve secretory *β*-gal-Pw production for both promoters after 32 h and 56 h of cultivation. Therefore, the optimized *β*-gal-Pw sequence was used for further experiments.

### Impact of cultivation temperature on *β*-gal-Pw secretion

**Fig. 3:**
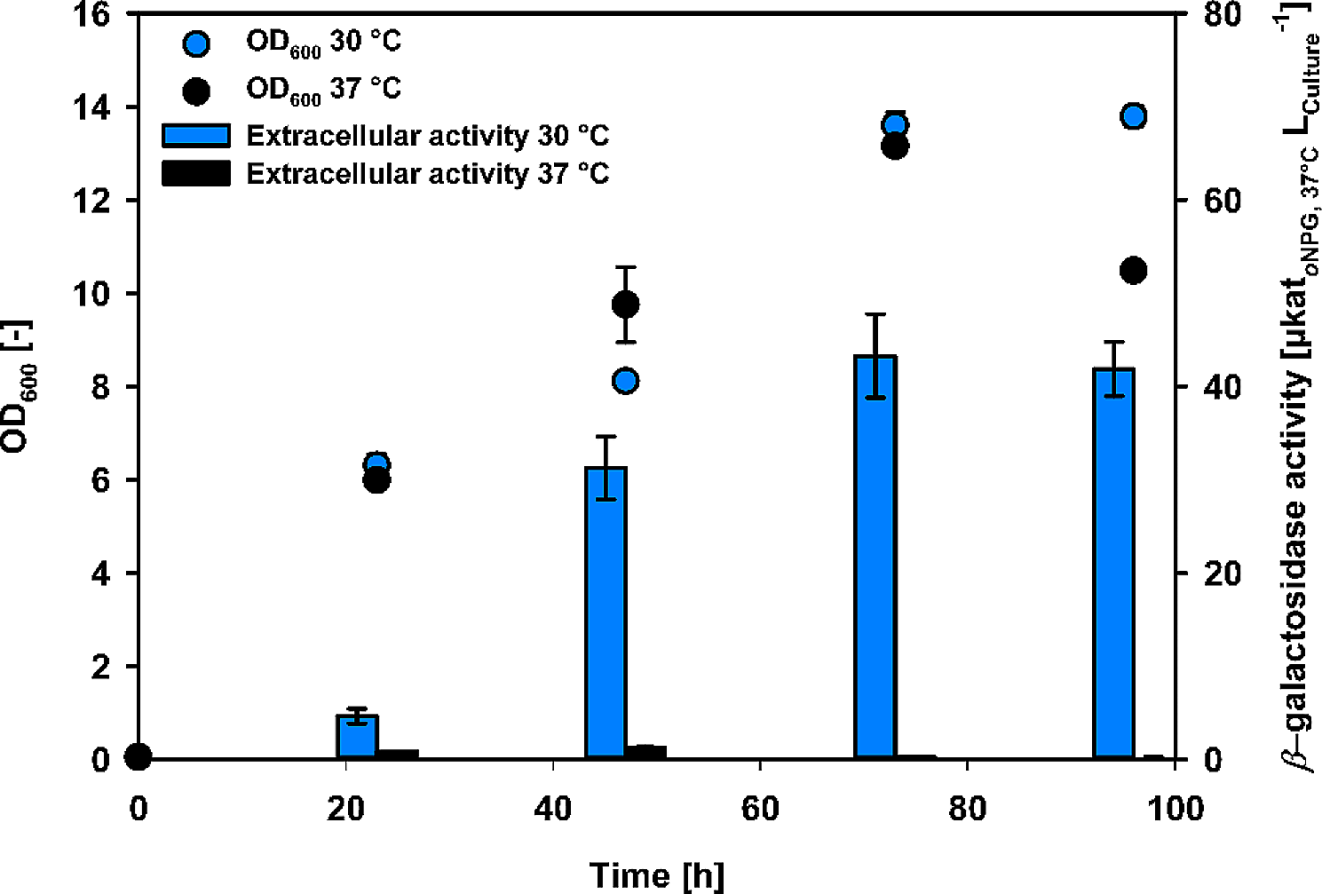
Shake flask cultivation of *Bs*43Y2 at 37 and 30 °C. Samples were taken after 24, 47, 73 and 96 h of cultivation

*Bs*43Y2 was cultivated at 30 and 37 °C to test the impact of the cultivation temperature on the *β*-gal-Pw secretion levels. The same pre-culture was used for the inoculation of all shake flasks. Surprisingly, extracellular *β*-galactosidase activity was significantly higher when cultivation was done at 30 °C (Fig. 3). The highest activity of 43.3 ± 5 µkat/L was reached after 73 h of cultivation, whereas only 1.3 µkat/L were detected extracellularly for cultivation at 37 °C. However, the growth behavior was similar at both temperatures over 73 h of cultivation. Therefore, it can be concluded that the higher cultivation temperature is unfavorable for *β*-gal-Pw secretion or *β*-gal-Pw functionality in the culture supernatant.

### Combinatorial screening of different signal peptides and promoters increases secretory production

**Fig. 4:**
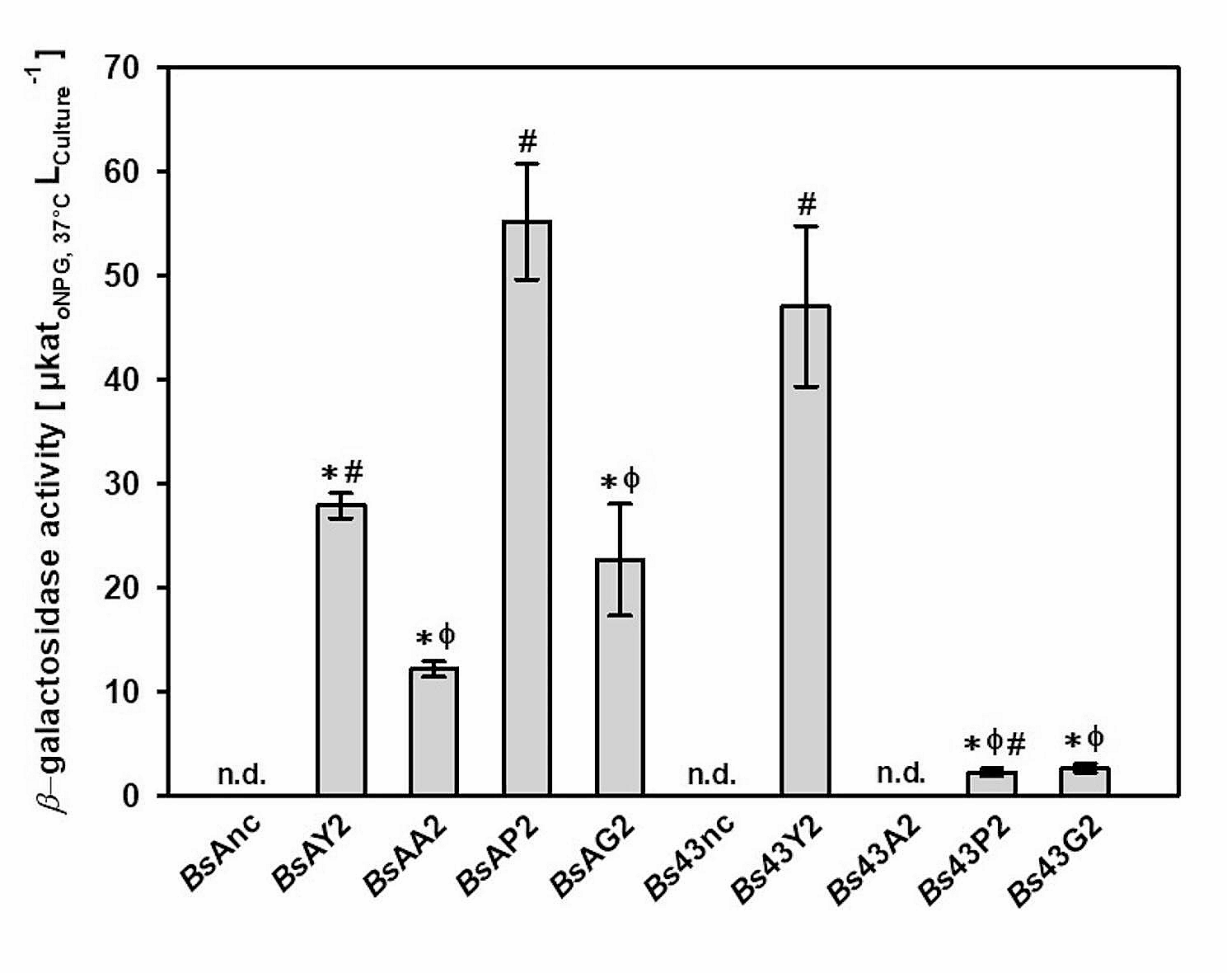
Combinatorial screening of different promoters and signal peptides for *β*-gal-Pw production. Different promoters (*Bs*A = P_AprE_ and *Bs*43 = P_43_) and signal peptides (Y = YoaW; A = AprE; G = GlmU; P = PhoD; nc = negative control, empty vector) were tested. The *β*-galactosidase activity was determined in the supernatant after 73 h of cultivation. Significance analyses between the activities of all strains were performed and significance (*p* < 0.05) is indicated by: * = significance to *Bs*43Y2; Φ = significance to *Bs*AP2; # = significance to *Bs*AA2. n.d. = activity not detected

The two well-described P_AprE_ and P_43_ promoters were tested with four different signal peptides fused to the codon-optimized *β*-gal-Pw gene to further improve the secretion of *β*-gal-Pw (Fig. 1). The YoaW (Y) and the AprE (A) signal peptide were tested, which mediate secretion via the Sec pathway. Furthermore, secretion by the TAT signal peptides GlmU (G) and PhoD (P) was investigated. The different recombinant *B. subtilis* strains were cultivated for 73 h at 30 °C and the extracellular *β*-galactosidase activity was determined. Signal peptides of the Sec and the TAT pathway mediated *β*-gal-Pw secretion, demonstrating the export of the unfolded and folded enzyme, respectively. The highest extracellular activity of 55.2 ± 6 µkat/L was detected for *Bs*AP2 with the P_AprE_ promoter and PhoD signal peptide (Fig. 4). Nevertheless, significant differences were observed between the different combinations, demonstrating the need for a combinatorial screening of promoters and signal peptides. The P_43_ promoter showed a high production yield of 47.0 ± 8 µkat/L only in combination with the YoaW signal peptide in *Bs*43Y2, whereas only low extracellular activities of less than 2.7 µkat/L were detected using the other signal peptides. By contrast, the extracellular activities were different when the expression was controlled by the P_AprE_ promoter. Here, the overall secretion yield was higher. Extracellular activities of 27.9 ± 1 and 22.7 ± 5 µkat/L were detected with the YoaW and GlmU signal peptide in *Bs*AY2 and *Bs*AG2, respectively, while *Bs*AA2 showed less activity of 12.2 ± 1 µkat/L. In addition, extracellular *β*-galactosidase activity was not observed for the negative control strains *Bs*Anc and *Bs*43nc, demonstrating that the activity measured can be attributed to the *β*-gal-Pw. These differences in secretion were underlined by SDS PAGE (Additional File 1 Figure S2).

### Correct processing of the secreted *β*-gal-Pw

After translocation via the Sec and the TAT pathway, the N-terminal signal peptide is cleaved by signal peptidases [54, 55]. Analyses of the intracellular proteome and the secretome were done to verify the cleavage of the signal peptide during translocation indicating proper secretion of the *β*-gal-Pw. The SDS PAGE analyses were done for *Bs*AP2, which showed the highest extracellular *β*-galactosidase activity in the screening experiments. The secretome and intracellular proteome were visualized during cultivation and a 120 kDa *β*-gal-Pw band was detected (Additional File 1: Figure S4). The respective bands of the secreted and the intracellular *β*-gal-Pw were analyzed by mass spectrometry (MS). The PhoD signal peptide fused to the *β*-gal-Pw was detected for the intracellular sample (Additional File 1: Figure S5A). By contrast, the signal peptide was not found within the secreted *β*-gal-Pw sequence (Additional File 1: Figure S5B). Consequently, the absence of the signal peptide in the secretome indicated the signal peptide mediated *β*-gal-Pw secretion via the TAT pathway.

### Bioreactor cultivation for secretory *β*-galactosidase production

**Fig. 5:**
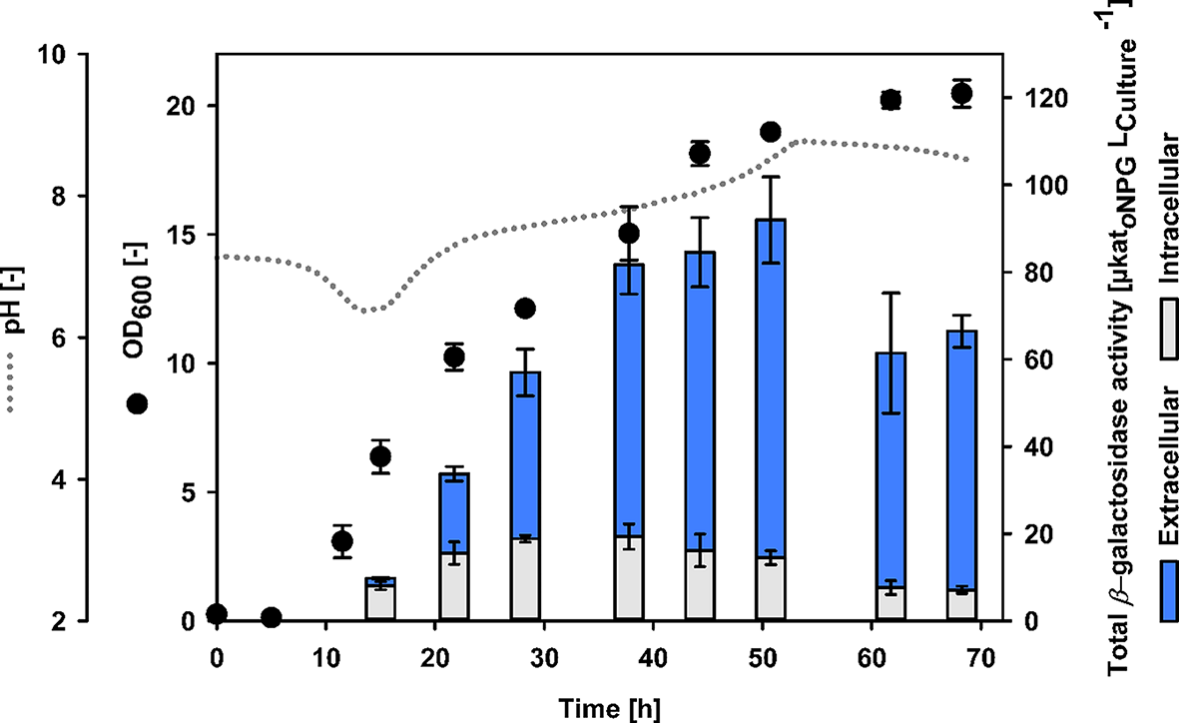
Cultivation of *Bs*AP2 in 1 L bioreactors for secretory *β*-gal-Pw production. Cultivation was done in duplicates at 30 °C, without pH control and with a working volume of 0.8 L

*Bs*AP2 was found to reach the highest extracellular *β*-galactosidase activity of 55.2 ± 6 µkat/L in the screening experiments. Therefore, this strain was cultivated in 1 L bioreactors (Fig. 5; Additional File 1 Figure S3).

**Fig. 6:**
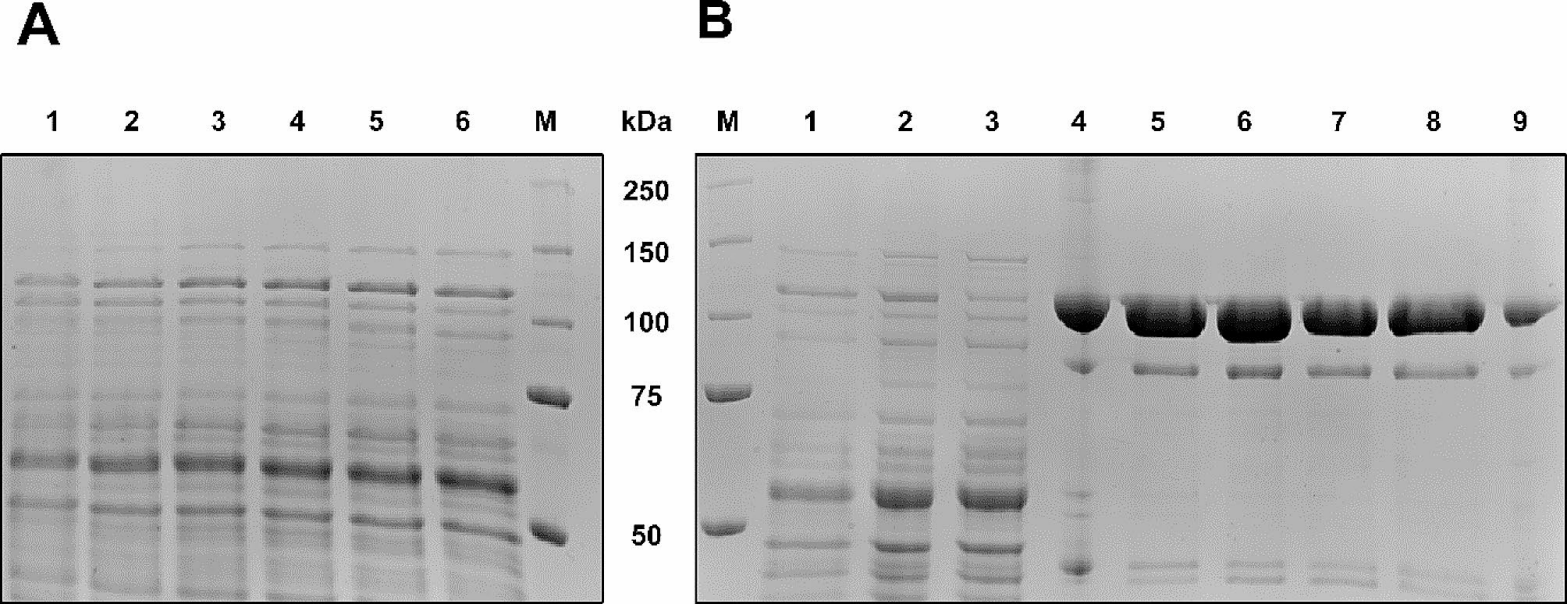
SDS PAGE of (**A**) secretome and (**B**) partial *β*-gal-Pw purification from the supernatant of *Bs*AP2 bioreactor cultivation. (**A**) The culture supernatant was analyzed after 1 = 22 h, 2 = 28 h, 3 = 38 h, 4 = 44 h, 5 = 51 h and 6 = 68 h of cultivation. (**B**) 1 = Supernatant of cultivation; 2 = Concentrated supernatant; 3 = Flow-through fraction IMAC; 4–9 = Fractions of IMAC purification. An amount of 8 µg of protein was loaded in each lane

Growth increased continuously until 62 h of cultivation, reaching the highest OD_600_ of 20 (corresponding to a bio dry mass of ~ 11 g L^− 1^). The pH was not regulated, showing a pH drop from 7.2 to 6.4 when growth started. Afterwards, the pH increased continuously to 8.5. This characteristic pH pattern has already been described for growth in complex soy medium [e.g. 56–58] and was found in the shake flask cultivations as well. Simultaneously, extracellular *β*-galactosidase activity increased during cultivation (Fig. 5). A maximum of 77.5 ± 10 µkat/L was reached after 51 h, which was 1.4-fold higher than in the shake flask experiments. This result correlates with the increasing intensity of the corresponding band (~ 120 kDa) on SDS PAGE (Fig. 6A).

The comparison of intra- and extracellular *β*-galactosidase activity during the bioreactor cultivation indicates an efficient secretion (Fig. 5). The intracellular activity increased at the beginning of the cultivation, remained at a similar level of 16 to 19 µkat/L from 22 until 44 h and slightly decreased at the end of cultivation. Consequently, the secretion efficiency increased from 55% after 22 h to almost 90% at the end of cultivation.

The culture supernatant of the bioreactor cultivations (Fig. 5) was pooled and concentrated using a 30 kDa cutoff filtration cassette and purified by IMAC. A purification factor of 16 was reached with a yield of 41% (Table 1). This corresponds to a total yield of 21 mg purified *β*-gal-Pw per 1 L supernatant. In addition, concentrating and purification steps were visualized by SDS PAGE (Fig. 6B). A clear 120 kDa band for the purified *β*-gal-Pw was observed. The band was analyzed by MS showing 92% sequence coverage with the *β*-gal-Pw, whereby only the PhoD signal peptide was not detected (Additional File 1: Figure S5C). In addition, the IMAC purified *β*-gal-Pw was analyzed by size exclusion chromatography to investigate whether the enzyme exists as a mono- or oligomer. A dominant peak was detected (Additional File 1: Figure S6), which correlates to an estimated molecular weight of ~ 100 kDa. Therefore, the size exclusion chromatography analysis showed that the secreted and purified *β*-gal-Pw resulted in an enzyme preparation containing a homogenous solution of monomeric *β*-gal-Pw.

**Table 1:** Purification table of IMAC purification of *β*-gal-Pw from the supernatant after *Bs*AP2 reactor cultivation. EA = Enzyme activity

Sample	Volume [mL]	Vol. EA [nkat/mL]	Spec. EA [nkat/mg]	Protein [mg/mL]	Total EA [nkat]	Yield %	Purification factor
Supernatant	463.1	37.5	42.2	0.9	17368.3	100	1.00
Load	49.5	298.3	40	7.5	14766.8	85	1.5
Elution	12	593.1	682.1	0.9	7117.7	41	16.2

## Discussion

The cytoplasmic 120 kDa *β*-gal-Pw was efficiently secreted with a recombinant *B. subtilis* strain for the first time, while attempts to secrete the *β*-gal-Pw in yeasts had limited success. The yeasts *Komagataella phaffii* or *Yarrowia lipolytica* provide a high secretion capacity and are often applied for secretory enzyme production [59, 60]. However, *β*-gal-Pw secretion was not possible using *K. phaffii* testing eleven different signal peptides and only barely successful with *Y. lipolytica* (unpublished observations). The main bottleneck of secretion in yeasts is the retention of the recombinant protein in the endoplasmic reticulum and degradation via the endoplasmic reticulum-associated protein degradation pathway [8]. By contrast, using prokaryotic hosts, such as *B. subtilis*, these eukaryotic-specific bottlenecks are bypassed due to different secretion mechanisms. Thus, testing prokaryotic and eukaryotic production hosts demonstrates big differences in production yield and highlights the importance of investigating various organisms for the production of a specific target enzyme.

Nevertheless, differences in secretory *β*-gal-Pw production with *B. subtilis* were also revealed when cultivation was done at different temperatures (Fig. 3). Yang et al. tested the impact of temperature on promoter activity and found a decrease in the P_43_ promoter activity when the temperature was reduced from 37 to 30 °C [61]. However, the yield of extracellular *β*-gal-Pw in this study was significantly lower at 37 °C. Temperature generally profoundly impacts the cell metabolism and, consequently, affects not only gene expression but also other steps in the production process, such as post-translational folding or protein stability in the supernatant. Therefore, higher cultivation temperatures negatively impact the production of extracellular *β*-gal-Pw and finally lead to a reduction of active *β*-gal-Pw. A lower cultivation temperature seemed to be beneficial for the *β*-gal-Pw production process with *B. subtilis* has also been shown for other enzymes [62, 63].

Secretion of the *β*-gal-Pw with *B. subtilis* was facilitated by signal peptides of both the Sec and the TAT secretion pathways. However, the highest extracellular activity was observed with *Bs*AP2, which possessed the PhoD signal peptide (Fig. 4). The *β*-gal-Pw secretion via the TAT pathway is consistent with the results of other studies in which *β*-galactosidases were efficiently secreted with *B. subtilis* using TAT signal peptides [15, 29, 49].

A total of two promoters were compared in combination with four different signal peptides for *β*-gal-Pw secretion with *B. subtilis* (Fig. 1). Both promoters, P_43_ and P_AprE_, have the highest activity at the transition and early stationary phase [45, 46]. However, the P_43_ promoter is often described to be significantly stronger compared to the P_AprE_ promoter [18, 64]. Contrarily, expression with the P_AprE_ resulted in a higher *β*-gal-Pw yield when using the signal peptides AprE, PhoD and GlmU (Fig. 4). The P_43_ promoter showed higher yields only when the YoaW signal peptide was fused to the *β*-gal-Pw. The P_AprE_ sequence applied possesses the 5’ untranslated region of the  *aprE* gene. This includes the  *aprE* leader sequence, forming an RNA hairpin structure, which stabilizes the mRNA significantly, resulting in a long mRNA half-life in the cell [65]. The  *aprE* leader sequence may be responsible for the higher stability of the *β*-gal-Pw mRNA and, consequently, results in a higher production yield.

In 2014, Wang et al. tested different combinations of signal peptides and promoters for pullulanase secretion. They found that the secretion efficiency of the signal peptides was independent of the promoter used [66]. Conversely, in this study, the efficient secretion was highly dependent on the specific combination of promoter and signal peptide. When the PhoD signal peptide was tested in combination with the two promoters (Fig. 4), *Bs*AP2 showed the highest *β*-gal-Pw yield of 55.2 µkat/L in shake flask experiments, whereas the secretory production with the P_43_ promoter (*Bs*43P2) was low with 2.2 µkat/L. In contrast, the highest yield using the P_43_ promoter was obtained with the YoaW signal peptide (*Bs*43Y2), which showed only moderate *β*-gal-Pw secretion in combination with the P_AprE_ promoter (*Bs*AY2). It is described that the 5’ end of the coding sequence together with the 5’ untranslated region can influence protein production post-transcriptionally e.g. due to the formation of mRNA secondary structures [67, 68]. Those structures around the ribosomal binding site can determine the translation rate [69] and consequently, affect production. It was speculated, that the contrasting results in this study might be due to post-transcriptional differences, such as translation initiation. Therefore, the theoretical translation initiation rates (TIR) were determined for the different constructs using the RBS calculator v2.1 [44]. The calculated TIR did not correlate with all the differences in secretion observed. However, the TIR for *Bs*43P2 possessing the P_43_ promoter combined with the PhoD signal peptide was noticeably lower than with all other signal peptides. This could indicate a possibly hampered translation initiation resulting in a significantly lower production yield compared to *Bs*AP2 (Additional File 1: Figure S7). Additionally, the high TIR for *Bs*43Y2 correlated with the maximum activity observed for this strain when the P_43_ promoter was used. Nevertheless, further experiments are required to reliably demonstrate the impact of translation initiation on *β*-gal-Pw secretion.

The secretory *β*-gal-Pw production by *Bs*AP2 was increased 1.4-fold and secretion efficiencies of more than 80% were achieved in a bioreactor cultivation. Similar secretion efficiencies with *B. subtilis* were found for the *β*-galactosidase Bgal1-3 from a genomic library. Here, the PhoD signal peptide was also used mediating Bgal1-3 secretion with 78% efficiency [49]. In addition, Xia et al. secreted the thermostable *β*-galactosidase from *Geobacillus stearothermophilus* (bgaB) using the PhoD signal peptide [15]. Secretion was less efficient but co-expression of TatAd and TatAc, which encode the TAT translocase, enhanced the secretory bgaB production. Both studies used *B. subtilis* strain 168 as a production host, while the extracellular protease deficient strain *B. subtilis* SCK6 [35] was used in this study. Due to the disruption of the two major extracellular proteases AprE and NprE, extracellular proteolysis is significantly reduced [34], suggesting that less extracellular degradation of the target protein is beneficial for higher secretion efficiency, as shown in various other studies [23, 48].

## Conclusion

Recombinant *B. subtilis* SCK6 successfully secreted the cytoplasmic, high molecular weight *β-*galactosidase of *P. wynnii*. Secretion was facilitated via Sec and TAT signal peptides with high efficiency of more than 80%. Combinatorial testing of promoters, signal peptides and codon optimization improved the secretory production and provides a basis for further optimizations of the production process. This study gave a straightforward approach for the secretion of a specific target enzyme and shows the potential of *B. subtilis* for the production of high molecular weight enzymes such as the *β-*gal-Pw.

### Electronic supplementary material

Below is the link to the electronic supplementary material.

Supplementary Material 1

## Data Availability

All data generated or analyzed during this study are included in this published article [and its additional file].
