# Supplementary material for: Secretion of the cytoplasmic and high molecular weight β-galactosidase of Paenibacillus wynnii with Bacillus subtilis
Source: Microb Cell Fact. 2024 Jun 12;23:170. doi: 10.1186/s12934-024-02445-7 (PMC11167759; doi:10.1186/s12934-024-02445-7)
Supplement: Supplementary file 1 — Supplementary Material 1 [file 12934_2024_2445_MOESM1_ESM.pdf]

Additional file 1

Secretion of the cytoplasmic and high molecular weight *β*-galactosidase of *Paenibacillus wynnii* with *Bacillus subtilis*

Jana Senger, Ines Seitl, Eva Pross, Lutz Fischer*

University of Hohenheim, Institute of Food Science and Biotechnology,

Department of Biotechnology and Enzyme Science, Garbenstr. 25, 70599 Stuttgart, Germany

*Corresponding author:

E-mail address: lutz.fischer@uni-hohenheim.de

Tel.: +49 711 459 22311

Keywords: *Bacillus subtilis,* protein secretion, recombinant enzyme production, *β*-galactosidase, bioreactor

Table S1: Oligonucleotides used in this study.

Name	Sequence (5'→3')
O1	ctagtgaaaaagatgttgatgttagcttttacatttcttttggctttgactatccatgtaggggg
O2	cgcgccccctacatggatagtcaaagccaaaagaaatgtaaaagctaacatcaacatctttttca
P1	cgtctcacgcgcaggcgcgtaaaaaacttgtctattcacc
P2	ccttgtctacaaaccccttaaaaacg
P3	tcgagtctctacggaaatagcg
P4	catcgataagctcaggcagtcaggatccg
P5	cctggaattcgccaaagctgtaatggctg
P6	gcactagtcattcctctcttacctataatgg
P7	cgtctcacgcgccgtaaaaaacttgtc
P8	gcctcgagcaccattctaatcgtaaatc

Table S2: Signal peptides used in this study.

Signal peptide	Amino acid sequence	Origin
YoaW	VKKMLMLAFTFLLALTIHVGGAQA	*B. subtilis* 168
AprE	VRSKKLWISLLFALALIFTMAFGSTSSAQA	*B. subtilis* 168
PhoD	VAYDSRFDEWVQKLKEESFQNNTFDRRK FIQGAGKIAGLSLGLTIAQSASA	*B. subtilis* 168
GlmU	VDRRDNGGQYMDKRFAVVLA	*B. licheniformis*

Table S3: Plasmids used in this study.

Plasmid	Application
Bs_Pgeo_SPaprE_prom pMA	Provides modular cloning cassette
20AC44OP_bGalPW_opt_Bs_pMK-RQ	Provides optimized *β*-gal-Pw gene
pET20b - *β*_Gal_ *P.wynnii*_His6	Provides native *β*-gal-Pw gene
BNspeBif3	Vector backbone
pLF_P_{43}_YoaW	Negative control
pLF_P_{AprE}_AprE	Negative control
pLF_ P_{43}_YoaW_ *β*-gal-Pw	*β*-gal-Pw expression
pLF_ P_{43}_YoaW_ *β*-gal-Pw_opt	*β*-gal-Pw expression
pLF_ P_{43}_AprE_ *β*-gal-Pw_opt	*β*-gal-Pw expression
pLF_ P_{43}_PhoD_ *β*-gal-Pw_opt	*β*-gal-Pw expression
pLF_ P_{43}_GlmU_ *β*-gal-Pw_opt	*β*-gal-Pw expression
pLF_P_{AprE}_YoaW_ *β*-gal-Pw	*β*-gal-Pw expression
pLF_P_{AprE}_YoaW_ *β*-gal-Pw_opt	*β*-gal-Pw expression
pLF_P_{AprE}_AprE_*β*-gal-Pw_opt	*β*-gal-Pw expression
pLF_P_{AprE}_PhoD_*β*-gal-Pw_opt	*β*-gal-Pw expression
pLF_ P_{AprE} GlmU_*β*-gal-Pw_opt	*β*-gal-Pw expression

Table S4: Strains used in this study.

Strain	Genotype and properties	Source
Escherichia coli XL1 Blue	*endA1 gyrA96* (nal^R) thi-1 *recA1 relA1 lac* glnV44 F'[:Tn10 proAB+ *lacIq* Δ(l*acZ*)M15] hsdR17(rK - mK +)	Bullock et al., 1987
Bacillus subtilis 168	*trpC2*	Burkholder & Giles, 1947
B. subtilis SCK6	Erm^R *, his nprR2 nprE18 ΔaprA3 ΔeglS102 ΔbglT bglSRV lacA*::P_{xylA} *-comK*	Zhang & Zhang, 2011
*Bs*Anc	*B. subtilis* SCK6 with plasmid pLF_P_{AprE}_AprE (negative control)	This work
*Bs*AY1	*B. subtilis* SCK6 with plasmid pLF_P_{AprE}_YoaW_*β*-gal-Pw	This work
*Bs*AY2	*B. subtilis* SCK6 with plasmid pLF_P_{AprE}_YoaW_*β*-gal-Pw_opt	This work
*Bs*AP2	*B. subtilis* SCK6 with plasmid pLF_P_{AprE}_PhoD_*β*-gal-Pw_opt	This work
*Bs*AG2	*B. subtilis* SCK6 with plasmid pLF_P_{AprE}_GlmU_*β*-gal-Pw_opt	This work
*Bs*AA2	*B. subtilis* SCK6 with plasmid pLF_P_{AprE}_AprE_*β*-gal-Pw_opt	This work
*Bs*43nc	*B. subtilis* SCK6 with plasmid pLF_P_{43}_YoaW (negative control)	This work
*Bs*43Y1	*B. subtilis* SCK6 with plasmid pLF_P_{43}_YoaW_*β*-gal-Pw	This work
*Bs*43Y2	*B. subtilis* SCK6 with plasmid pLF_P_{43}_YoaW_*β*-gal-Pw_opt	This work
*Bs*43P2	*B. subtilis* SCK6 with plasmid pLF_P_{43}_PhoD_*β*-gal-Pw_opt	This work
*Bs*43G2	*B. subtilis* SCK6 with plasmid pLF_P_{43}_GlmU_*β*-gal-Pw_opt	This work
*Bs*43A2	*B. subtilis* SCK6 with plasmid pLF_P_{43}_AprE_*β*-gal-Pw_opt	This work

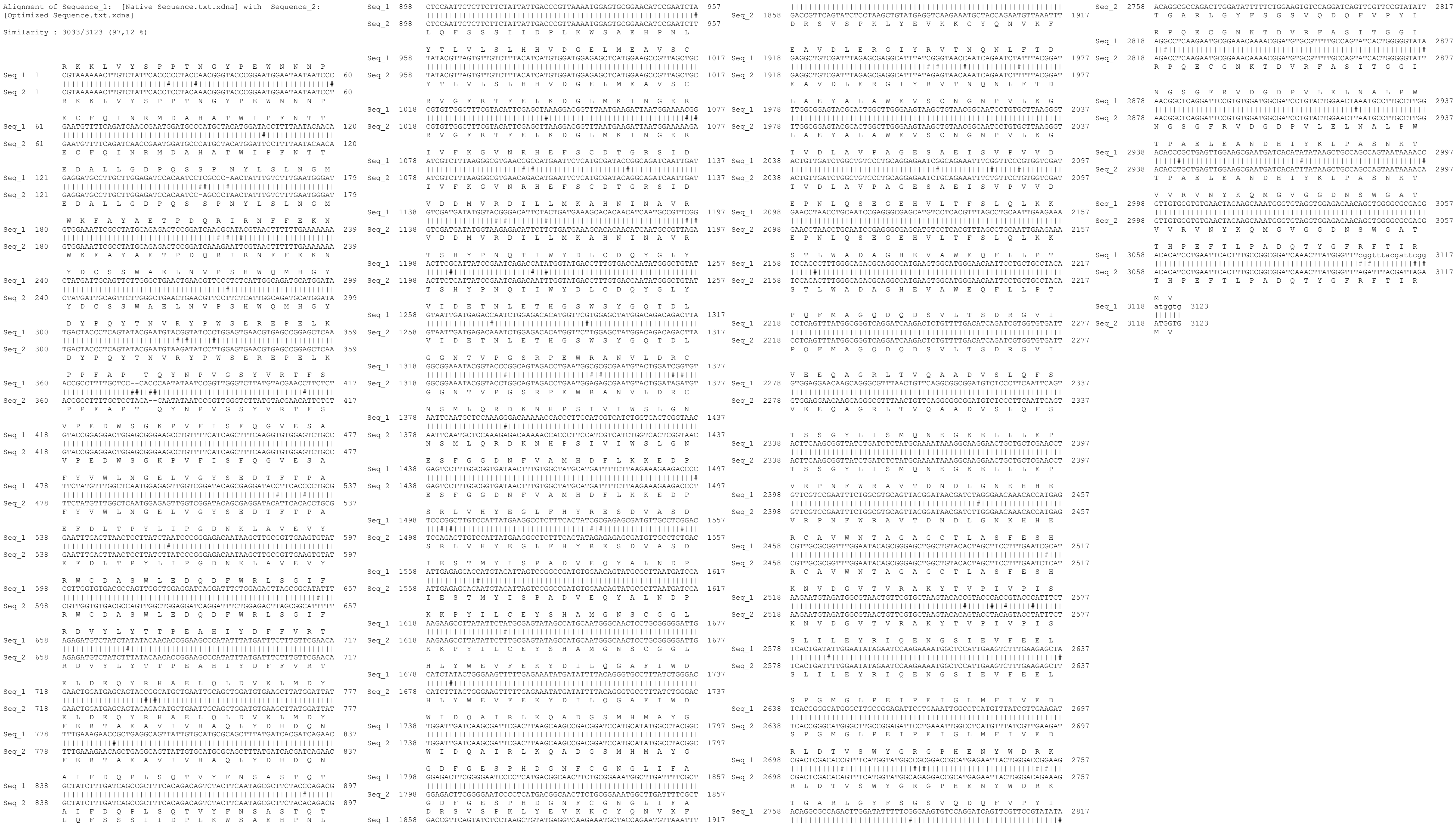

Figure S1: Alignment of native *β*-gal-Pw sequence (Seq1) and codon-optimized *β*-gal-Pw sequence (Seq2).

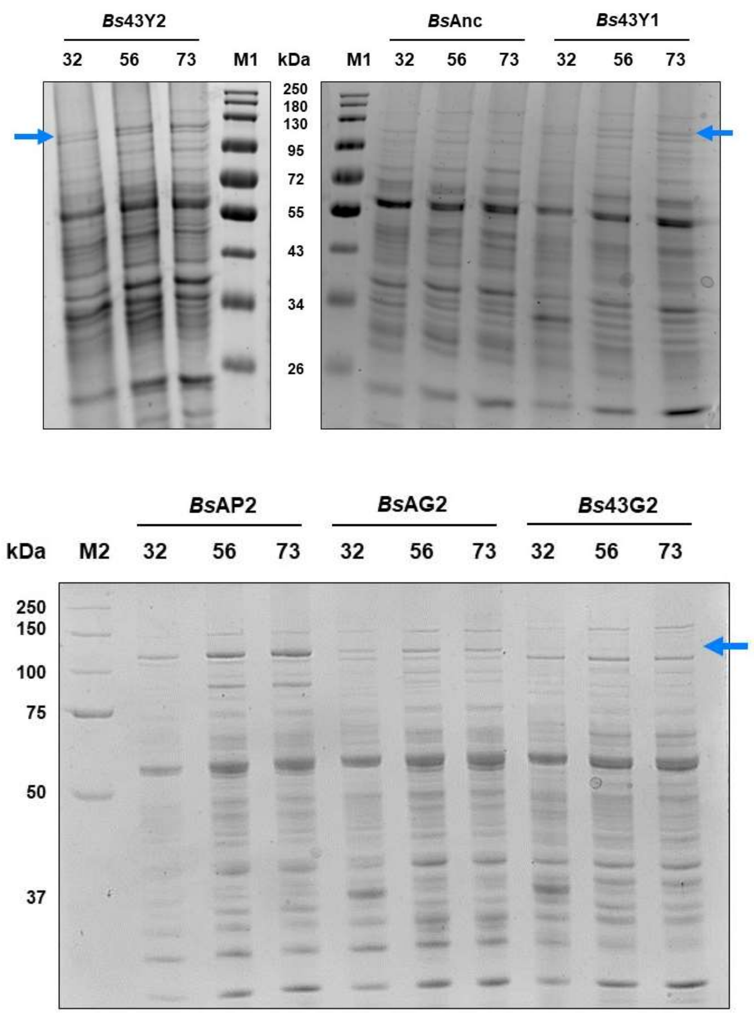

Figure S2: SDS PAGEs of the secretomes of the different *B. subtilis* strains during shake flask cultivation. The culture supernatant was analyzed after 32 h, 56 h and 73 h of cultivation. The blue arrow indicates the 120 kDa band corresponding to the size of the β-gal-Pw. An amount of 8 µg of protein was loaded in each lane. M1=Biorad Precision Plus Protein™ Unstained Protein Standards; M2=NEB Color Prestained Protein Standard Broad Range (10-250 kDa)

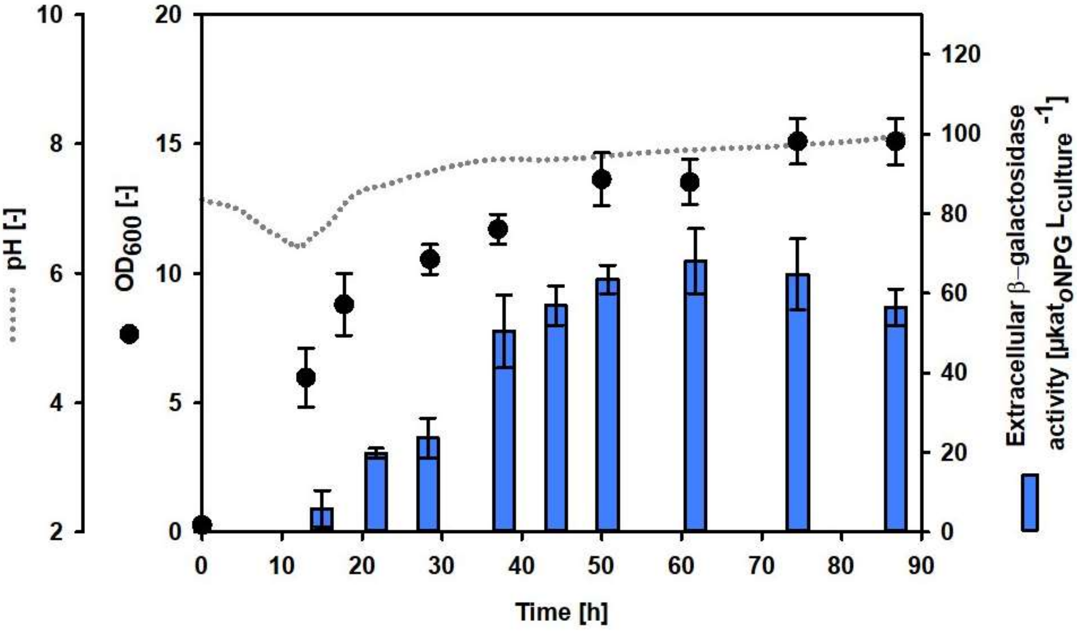

Figure S3: Repetition of the bioreactor cultivation of *Bs*AP2 for secretory *β*-gal-Pw production. Cultivation was done in duplicates at 30 °C, without pH control and with a working volume of 0.8 L.

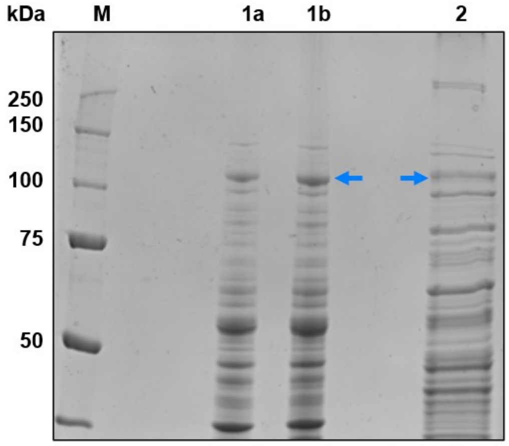

Figure S4: SDS PAGE of secretome (1) and intracellular proteome (2) of *Bs*AP2. The bands (blue arrow) corresponding to a size of 120 kDa were analyzed by MS. Supernatant and cell free extract after 73 h of shake flask cultivation were analyzed. The supernatant sample was loaded twice (1a and b). An amount of 8 % SDS gel was used and 8 µg of protein was loaded. M = Marker

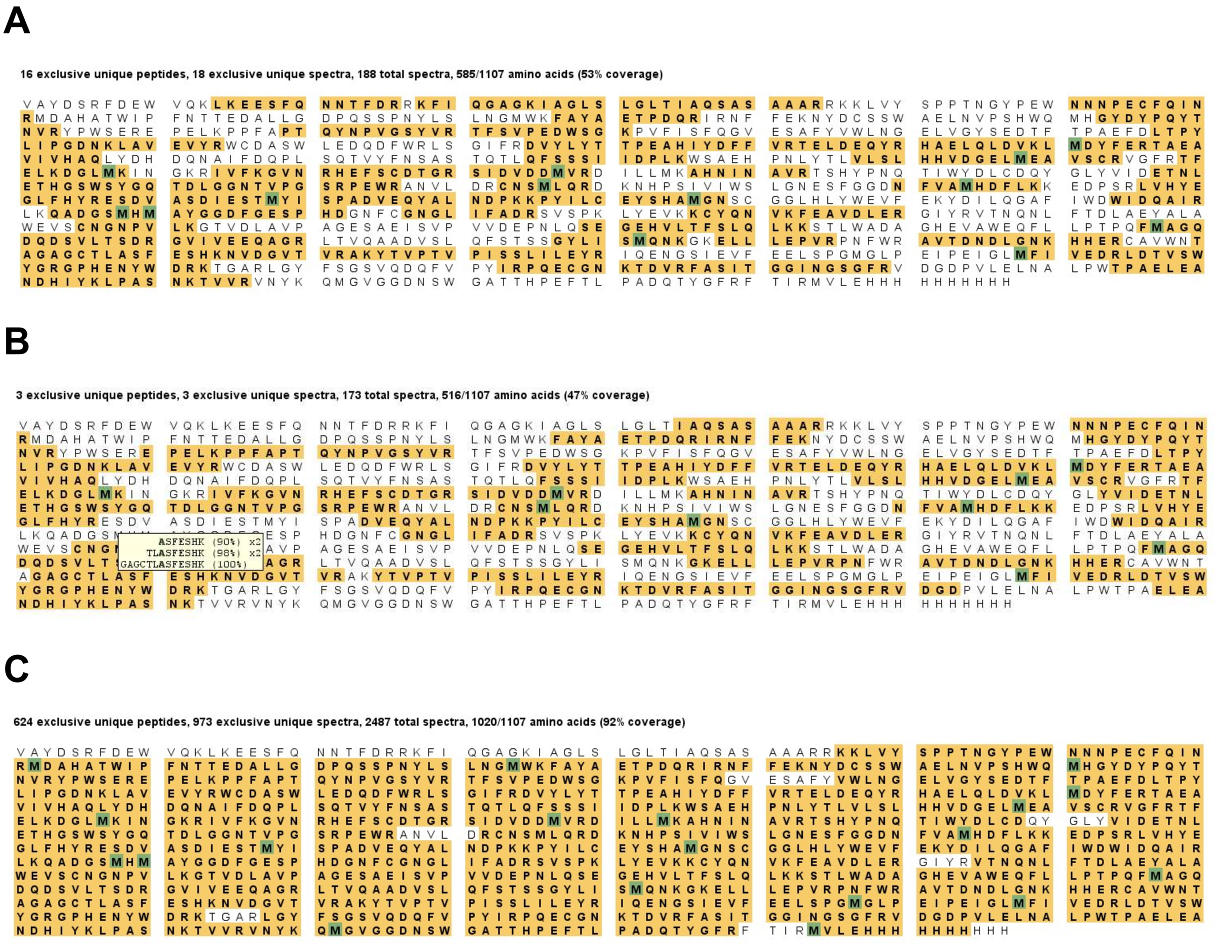

Figure S5: MS analyses results of (A) intracellular and (B) extracellular *β*-gal-Pw (C) purified *β*-gal-Pw. The *β*-gal-Pw in the supernatant of the bioreactor cultivation of *Bs*AP was purified.

A

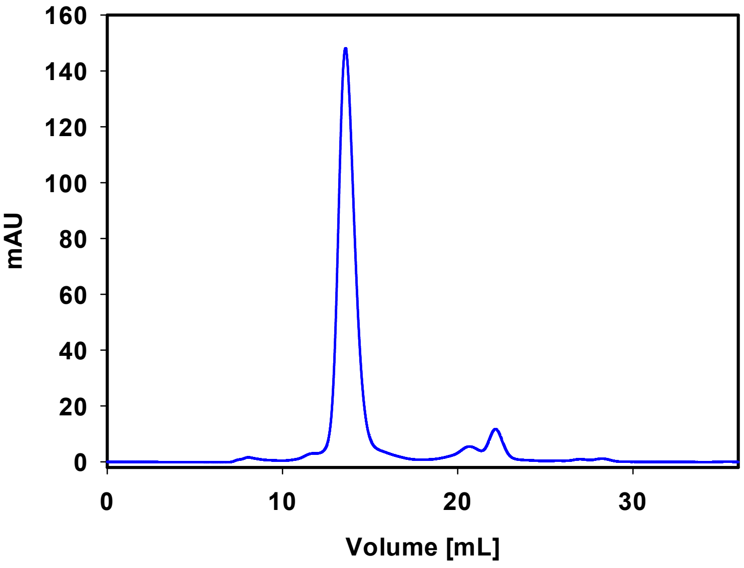

B

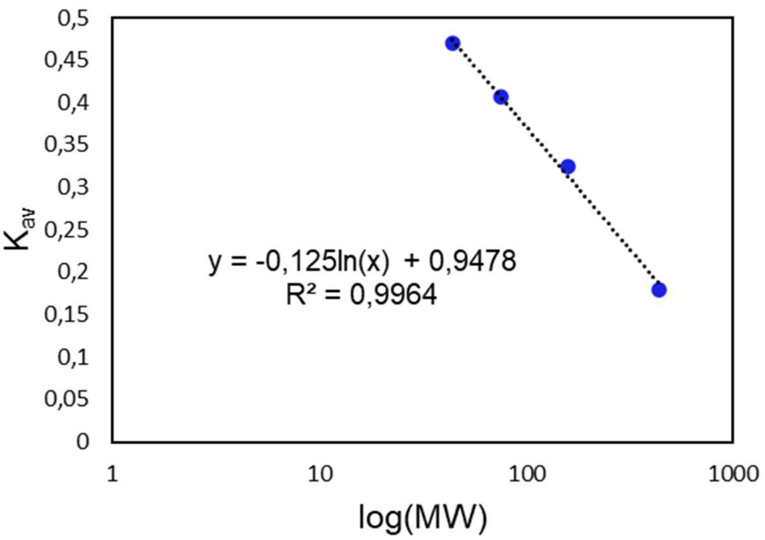

Figure S6: Chromatogram of size exclusion chromatography of purified *β*-gal-Pw (A) and the calibration curve generated (B). The *β*-gal-Pw was purified from the supernatant of the bioreactor cultivation of *Bs*AP2.

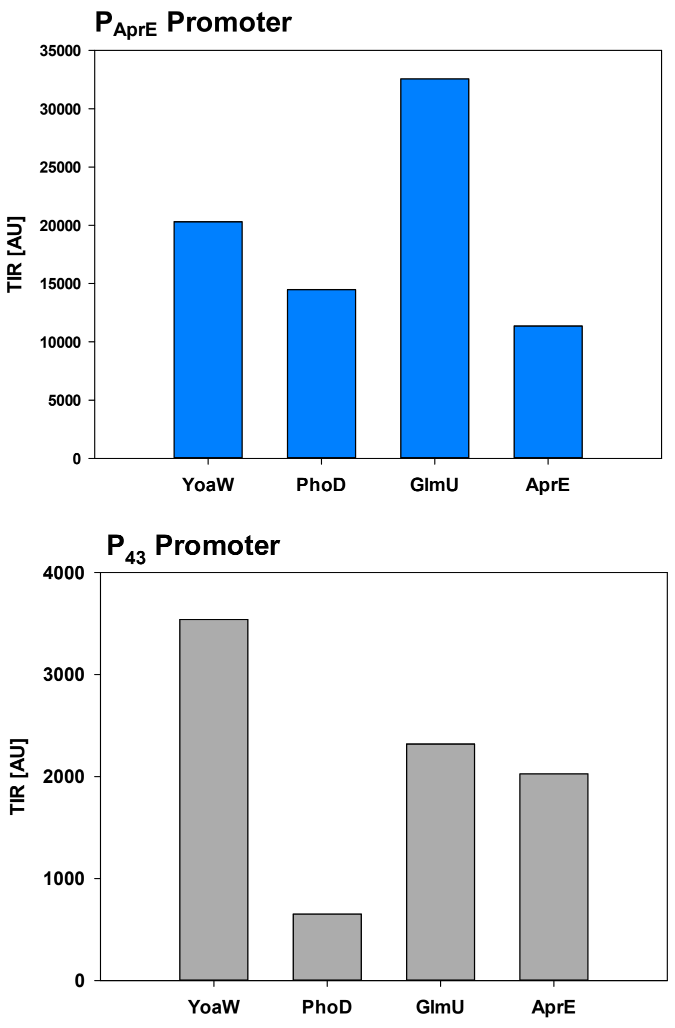

Figure S7: Translation initiation rates calculated with the RBS Calculator v2.1. for the different constructs. 100 nt upstream and 35 nt downstream of the start codon were considered. AU = Arbitrary units.

A

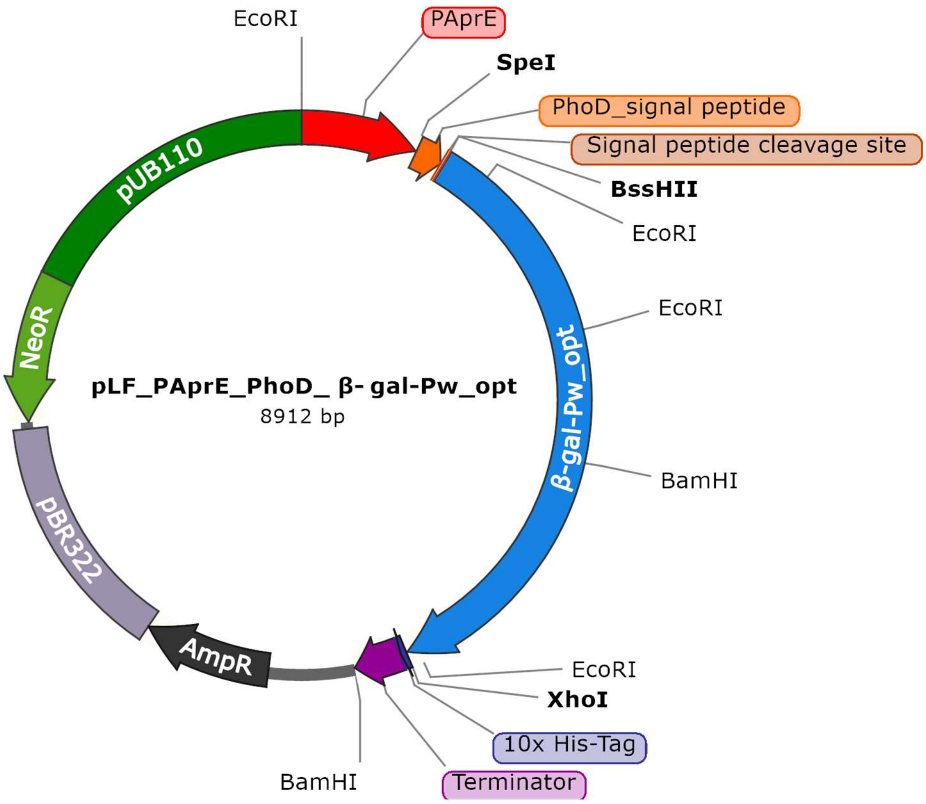

B

gaattcctccatttcttctgctatcaaaataacagactcgtgattttccaaacgagctttcaaaaaagcctctgccccttgc
aaatcggatgcctgtctataaaattcccgatattggttaaacagcggcgcaatggcggccgcatctgatgtctttgcttggcg
aatgttcatcttatttcttcctccctctcaataatttttcattctatcccttttctgtaaagtttatttttcagaatacttt
tatcatcatgctttgaaaaaatatcacgataatatccattgttctcacggaagcacacgcaggtcatttgaacgaatttttc
gacaggaatttgccgggactcaggagcatttaacctaaaaaagcatgacatttcagcataatgaacatttactcatgtctatt
ttcgttcttttctgtatgaaaatagttatttcgagtctctacggaaatagcgagagatgatatacctaaatagagataaaatc
atctcaaaaaaatgggtctactaaaatattattccatctattacaataaattcacagaatagtcttttaagtaagtctactct
gaattttttaaaaggagagggtaactagtggcatacgacagtcgttttgatgaatgggtacagaaactgaaagaggaaagct
ttcaaaacaatacgtttgaccgccgcaaatttattcaaggagcggggaagattgcaggactttctcttggattaacgattgcc
cagtcggctagcgccgcggcgcgccgtaaaaaacttgtctattcacctcctacaaacgggtacccggaatggaataataatcc
tgaatgttttcagatcaaccgaatggatgcccatgctacatggattccttttaatacaacagaggatgccttgcttggagatc
cacaatccagccctaactatttgtctttgaatgggatgtggaaattcgcctatgcagagactccggatcaaagaattcgtaac
tttttgaaaaaaactatgattgcagttcttgggctgaactgaacgttccttctcattggcagatgcatggatatgactaccc
tcagtatacgaatgtaagatatccttggagtgaacgtgagccggagctcaaaccgccttttgctcctacacaatataatccgg
ttgggtcttatgtacgaacattctctgtaccggaggactggagcgggaagcctgttttcatcagctttcaaggtgtggagtct
gccttctatgtttggctcaatggagagttggtcggatacagcgaggatacattcacacctgcggaatttgacttaactcctta
tcttatcccgggagacaataagcttgccgttgaagtgtatcgttggtgtgacgccagttggctggaggatcaggatttctgga
gacttagcggcattttagagatgtctatctttatacaacaccggaagcccatatttatgatttctttgttcgaacagaactg
gatgagcagtacagacatgctgaattgcagctggatgtgaagcttatggattattttgaaagaacagctgaggcagttattgt
gcatgcgcagctttatgatcacgatcagaacgctatctttgatcagccgctttcacagacagtctacttcaatagcgcttcta
cacagacgctccaattctcttcttctattattgacccgttaaaatggagtgcggaacatccgaatctttatacgttagtgttg
tctttacatcatgtggatggagagctcatggaagccgttagctgccgtgttggctttcgtacattcgagcttaaggacggttt
aatgaagattaatggaaaaagaatcgtctttaagggcgtgaacagacatgaattctcatgcgatacaggcagatcaattgatg
tcgatgatatggtaagagacattcttctgatgaaagcacacaacatcaatgccgttagaacttctcattatccgaatcagaca
atttggtatgacctttgtgaccaatatgggctgtatgtaattgatgagacaaatctggagacacatggttcttggagctatgg
acagacagacttaggcggaaatacggtacctggcagtagacctgaatggagagcgaatgtactggatagatgtaattcaatgc
tccaaagagacaaaaaccacccttccatcgtcatctggtcactcggtaacgagtcctttggcggtgataactttgtggctatg
catgattttcttaagaaagaagacccttccagacttgtccattatgaaggcctctttcactatagagagagcgatgttgcctc
tgacattgagagcacaatgtacattagtccggccgatgtggaacagtatgcgcttaatgatccaaagaagccttatattcttt
gcgagtatagccatgcaatgggcaactcctgcggggattgcatctttactgggaagtttttgagaaatatgatattttacag
ggtgcctttatctgggactggattgatcaagcgattcgacttaagcaagccgacggatccatgcatatggcctacggcggaga

cttcggggaatcccctcatgacggcaacttctgcggaaatggcttgattttcgctgaccgttcagtatctcctaagctgtatg
aggtcaagaaatgctaccagaatgttaaatttgaggctgtcgatttagagcgaggcatttatagagtaacaaatcagaatctt
tttacggatttggcggagtacgcactggcttgggaagtaagctgtaacggcaatcctgtgcttaagggtactgttgatctggc
tgtccctgcaggagaatctgcagaaatttctgttcctgtggtcgatgaacctaacctgcaatccgagggcgagcatgtcctca
cgtttagcctgcaattgaagaaatccacactttgggcagacgcaggccatgaagtggcatgggaacaattcctgctgcctaca
cctcagtttatggcgggtcaggatcaagactctgttttgacatcagatcgtggtgtgattgtggaggaacaagcagggcgttt
aactgttcaggcggcggatgtctcccttcaattcagtacttcaagcggttatctgatctctatgcaaaataaaggcaaggaac
tgctgctcgaacctgttcgtccgaatttctggcgtgcagttacggataacgatcttgggaacaaacaccatgagcgttgcgcg
gtttggaatacagcgggagctggctgtacactagcttcctttgaatctcataagaatgtagatggcgtaactgttcgtgctaa
gtacacagtacctacagtacctatttcttcactgattttggaatatagaatccaagaaaatggctccattgaagtctttgaag
agctttcaccgggcatgggcttgccggagattcctgaaattggcctcatgtttatcgttgaagatcgactcgacacagtttca
tggtatggcagaggaccgcatgagaattactgggacagaaagacaggcgccagacttggatatttttctggaagtgtccagga
tcagttcgttccgtatattagacctcaagaatgcggaaacaaaacggatgtgcgttttgccagtatcactggggggtattaacg
gctcaggattccgtgtggatggcgatcctgtactggaacttaatgccttgccttggacacctgctgagttggaagcgaatgat
cacatttataagctgccagccagtaataaaacagttgtgcgtgtgaactacaagcaaatgggtgtaggtggagacaacagctg
gggcgcgacgacacatcctgaattcactttgccggcggatcaaacttatgggtttagatttacgattagaatggtgctcgagc
accaccaccaccaccatcaccatcatcattaaacataaaaaaccggccttggccccgccggtttttttattatttttcttcctc
cgcatgttcaatccgctccataatcgacggatggctccctctgaaaattttaacgagaaacggcgggttgacccggctcagtc
ccgtaacggccaagtcctgaaacgtctcaatcgccgcttcccggtttccggtcagctcaatgccgtaacggtcggcggcgttt
tcctgataccgggagacggcattcgtaatcggatcctgactgcctgagcttatcgatgataagctgtcaaacatgagaattaa
ttcttgaagacgaaagggcctcgtgatacgcctatttttataggttaatgtcatgataataatggtttcttagacgtcaggtg
gcacttttcggggaaatgtgcgcggaacccctatttgtttatttttctaaatacattcaaatatgtatccgctcatgagacaa
taaccctgataaatgcttcaataatattgaaaaaggaagagtatgagtattcaacatttccgtgtcgcccttattcccttttt
tgcggcattttgccttcctgtttttgctcacccagaaacgctggtgaaagtaaaagatgctgaagatcagttgggtgcacgag
tgggttacatcgaactggatctcaacagcggtaagatccttgagagttttcgccccgaagaacgttttccaatgatgagcact
tttaaagttctgctatgtggcgcggtattatcccgtattgacgccgggcaagagcaactcggtcgccgcatacactattctca
gaatgacttggttgagtactcaccagtcacagaaaagcatcttacggatggcatgacagtaagagaattatgcagtgctgcca
taaccatgagtgataacactgcggccaacttacttctgacaacgatcggaggaccgaaggagctaaccgcttttttgcacaac
atgggggatcatgtaactcgccttgatcgttgggaaccggagctgaatgaagccataccaaacgacgagcgtgacaccacgat
gcctgtagcaatggcaacaacgttgcgcaaactattaactggcgaactacttactctagcttcccggcaacaattaatagact
ggatggaggcggataaagttgcaggaccacttctgcgctcggcccttccggctggctggtttattgctgataaatctggagcc
ggtgagcgtgggtctcgcggtatcattgcagcactggggccagatggtaagccctcccgtatcgtagttatctacacgacggg
gagtcaggcaactatggatgaacgaaatagacagatcgctgagataggtgcctcactgattaagcattggtaactgtcagacc
aagtttactcatatatactttagattgatttaaaacttcatttttaatttaaaaggatctaggtgaagatcctttttgataat
ctcatgaccaaaatcccttaacgtgagttttcgttccactgagcgtcagaccccgtagaaaagatcaaaggatcttcttgaga
tcctttttttctgcgcgtaatctgctgcttgcaaacaaaaaaaccaccgctaccagcggtggtttgtttgccggatcaagagc
taccaactctttttccgaaggtaactggcttcagcagagcgcagataccaaatactgtccttctagtgtagccgtagttaggc
caccacttcaagaactctgtagcaccgcctacatacctcgctctgctaatcctgttaccagtggctgctgccagtggcgataa
gtcgtgtcttaccgggttggactcaagacgatagttaccggataaggcgcagcggtcgggctgaacggggggttcgtgcacac
agcccagcttggagcgaacgacctacaccgaactgagatacctacagcgtgagctatgagaaagcgccacgcttcccgaaggg
agaaaggcggacaggtatccggtaagcggcagggtcggaacaggagagcgcacgagggagcttccagggggaaacgcctggta
tctttatagtcctgtcgggtttcgccacctctgacttgagcgtcgatttttgtgatgctcgtcaggggggcggagcctatgga
aaaacgccagcaacgcggcctttttacggttcctggccttttgctggccttttgctcacatgttctttcctgcgttatcccct
gattctgtggataaccgtattaccgcctttgagtgagctgataccgctcgccgcagccgaacgaccgagcgcagcgagtcagt
gagcgaggaagcggaagagcgcctgatgcggtattttctccttacgcatctgtgcggtatttcacaccgcatatggtgcactc
tcagtacaatctgctctgatgccgcatagttaagccagtatacactccgctatcgctacgtgactgggtcatggctgcgcccc
gacacccgccaacacccgctgacgcgccctgacgggcttgtctgctcccggcatccgcttacagacaagctgtgaccgtctcc
gggagctgcatgtgtcagaggttttcaccgtcatcaccgaaacgcgcgaggcaggtaaccaacatgattaacaattattagag
gtcatcgttcaaaatggtatgcgttttgacacatccactatatatccgtgtcgttctgtccactcctgaatcccattccagaa
attctctagcgattccagaagtttctcagagtcggaaagttgaccagacattacgaactggcacagatggtcataacctgaag
gaagatctgattgcttaactgcttcagttaagaccgaagcgctcgtcgtataacagatgcgatgatgcagaccaatcaacatg
gcacctgccattgctacctgtacagtcaaggatggtagaaatgttgtcggtccttgcacacgaatattacgccatttgcctgc
atattcaaacagctcttctacgataagggcacaaatcgcatcgtggaacgtttgggcttctaccgatttagcagtttgataca
ctttctctaagtatccacctgaatcataaatcggcaaaatagagaaaaattgaccatgtgtaagcggccaatctgattccacc
tgagatgcataatctagtagaatctcttcgctatcaaaattcacttccaccttccactcaccggttgtccattcatggctgaa
ctctgcttcctctgttgacatgacacacatcatctcaatatccgaatagggcccatcagtctgacgaccaagagagccataaa
caccaatagccttaacatcatccccatatttatccaatattcgttccttaatttcatgaacaatcttcattctttcttctcta
gtcattattattggtccattcactattctcattcccttttcagataattttagatttgcttttctaaataagaatatttggag
agcaccgttcttattcagctattaataactcgtcttcctaagcatccttcaatccttttaataacaattatagcatctaatct
tcaacaaactggcccgtttgttgaactactctttaataaaataatttttccgttcccaattccacattgcaataatagaaaat
ccatcttcatcggctttttcgtcatcatctgtatgaatcaaatcgccttcttctgtgtcatcaaggtttaatttttttatgtat
ttcttttaacaaaccaccataggagattaaccttttacggtgtaaaccttcctccaaatcagacaaacgtttcaaattctttt
cttcatcatcggtcataaaatccgtatcctttacaggatattttgcagtttcgtcaattgccgattgtatatccgatttatat
ttatttttcggtcgaatcatttgaacttttacatttggatcatagtctaatttcattgcctttttccaaaattgaatccattg
tttttgattcacgtagttttctgtattcttaaaataagttggttccacacataccaatacatgcatgtgctgattataagaat
tatctttattatttattgtcacttccgttgcacgcataaaaccaacaagattttttattaattttttttatattgcatcattcgg
cgaaatccttgagccatatctgacaaactcttatttaattcttcgccatcataaacatttttaactgttaatgtgagaaacaa
ccaacgaactgttggcttttgtttaataacttcagcaacaaccttttgtgactgaatgccatgtttcattgctctcctccagt
tgcacattggacaaagcctggatttacaaaaccacactcgatacaactttctttcgcctgtttcacgattttgtttatactct

aatatttcagcacaatcttttactctttcagccttttttaaattcaagaatatgcagaagttcaaagtaatcaacattagcgat
tttcttttctctccatggtctcacttttccactttttgtcttgtccactaaaacccttgatttttcatctgaataaatgctac
tattaggacacataatattaaaagaaacccccatctatttagttatttgtttagtcacttataactttaacagatggggtttt
tctgtgcaaccaattttaagggttttcaatactttaaaacacatacataccaacacttcaacgcacctttcagcaactaaaat
aaaaatgacgttatttctatatgtatcaagataagaaagaacaagttcaaaaccatcaaaaaaagacaccttttcaggtgctt
tttttattttataaactcattccctgatctcgacttcgttctttttttacctctcggttatgagttagttcaaattcgttctt
tttaggttctaaatcgtgtttttcttggaattgtgctgttttatcctttaccttgtctacaaaccccttaaaaacgtttttaa
aggcttttaagccgtctgtacgttccttaag

Figure S8: Plasmid map (A) and DNA sequence (B) of plasmid pLF_P_{AprE}_PhoD_*β*-gal-Pw_opt.

References:

Bullock WO, Fernandez JM, Short JM. XL1-Blue—a high-efficiency plasmid transforming *recA Escherichia coli* strain with *β*-galactosidase selection, Biotechniques 1987;5(3),376-379.

Burkholder PR, Giles NH. Induced biochemical mutations in *Bacillus subtilis*. Am. J. Bot. 1947;33:345–348.

Zhang XZ, Zhang YHP. Simple, fast and high-efficiency transformation system for directed evolution of cellulase in *Bacillus subtilis*. Microb Biotechnol. 2011;4:98-105.
